# Bio-inspired ultrasonic microreactor for efficient synthesis of indigo-emitting carbon dots with tunable morphology and enhanced optical properties

**DOI:** 10.1016/j.ultsonch.2025.107576

**Published:** 2025-09-17

**Authors:** Longshi Rao, Shengxin Zhu, Jiaying Liu, Qiuling Bai, Junxian Zou, Chuheng Deng, Hongze Tu, Qingxian Liu, Guisheng Zhong, Xiaodong Niu, Jiasheng Li

**Affiliations:** a Department of Mechanical Engineering, College of Engineering, Shantou University, Shantou 515063, China; b Intelligent Manufacturing Key Laboratory of Ministry of Education, Shantou University, Shantou 515063, China; c Guangdong Provincial Key Laboratory of Automotive Display and Touch Technologies, Shantou Goworld Display Technology Co., Ltd., Shantou 515041, China; d Shantou Key Laboratory for Intelligent Equipment and Technology, Shantou University, Shantou 515063, China; e National & Local Joint Engineering Research Center of Semiconductor Display and Optical Communication Devices, South China University of Technology, Guangzhou 510641, China

**Keywords:** Carbon dots, Ultrasonic microreactor, Bionic design, Numerical simulations, Process optimization

## Abstract

Carbon dots (CDs) are promising fluorescent nanomaterials with broad applications in optoelectronics, healthcare, and artificial photosynthesis. However, synthesizing indigo-emitting CDs with optimal properties remains challenging due to the inefficiencies and complexity of conventional methods. This study introduces a high-performance ultrasonic microreactor inspired by biomimetic leaf vein structures to enhance CD synthesis efficiency. We developed a COMSOL Multiphysics-based optimization framework to improve flow field uniformity and examine transport dynamics within the microchannel. This framework identified key parameters, including leaf vein contours, fractal angles, depth-to-width ratios, and inlet configurations, that govern flow characteristics. Additionally, we optimized ultrasonic energy transfer by directly coupling the transducer with the microreactor, determining the optimal frequency (21 kHz) and power (100 W) for maximum reactor performance. Visualization experiments revealed how ultrasound regulates bubble dynamics, enhancing interfacial area and stabilizing suspension behavior. Using this optimized system, we synthesized indigo-emitting CDs with a maximum PLQY of 27.5 % and a narrow FWHM of ∼78 nm under 365 nm excitation. Multivariate experiments revealed how flow velocity, reaction temperature, and ultrasonic modulation influence the optical properties of the CDs. This work underscores the synergistic combination of ultrasonic energy, biomimetic design, and simulation-guided optimization, providing a solid foundation for scalable synthesis of carbon-based nanomaterials with applications in optoelectronics, healthcare, and beyond.

## Highlights

• The leaf vein-inspired ultrasonic microreactor facilitates the synthesis of indigo-emitting CDs with tunable properties.
• A simulation-optimization framework was developed to identify key structural parameters, improving fluid dynamics.
• Visualization experiments elucidate the mechanism by which ultrasound regulate bubble dynamics within the microchannel.
• Multivariate coupling experiments reveal the PL spectra evolution of CDs, providing insights into their optical behavior.

## 1 Introduction

Carbon dots (CDs), a class of emerging and highly promising fluorescent carbon-based nanomaterials, have attracted significant attention across diverse fields due to their remarkable properties, such as excellent photothermal stability, size tunability, and good biocompatibility [[1], [2], [3], [4]]. However, current research on CDs has predominantly focused on the blue to red emission, with limited exploration of the indigo light (blue-violet, 420–450 nm) region [[5], [6], [7], [8], [9]]. Compared to conventional blue and green CDs, indigo-emissive CDs exhibit superior photostability, reduced photobleaching effects, and broad-spectrum emission, granting them distinct advantages in optoelectronics [10], healthcare [11], and artificial photosynthesis [12,13] applications. Despite their potential, traditional synthesis methods for indigo-emissive CDs (e.g., hydrothermal, solvothermal, and laser-assisted methods) typically require high temperature and pressure conditions, the use of expensive precursors or catalysts, and extended reaction times [[14], [15], [16]]. These factors not only increase the cost of the preparation process but also limit the feasibility of large-scale production. Consequently, the development of green chemical methods, simplification of the synthesis process, and the discovery of new synthetic routes to reduce costs are critical for advancing the widespread application of indigo-emitting CDs.

Microreactors, as precision-controlled reaction devices, offer significant advantages in enhancing reaction rates and improving particles uniformity, facilitating the production of high-quality CDs [[17], [18], [19]]. These advantages are especially apparent in the precise control of reaction parameters such as flow velocity, temperature, and pressure. The small volume of microreactors not only ensures uniform-sized CDs but also minimizes reactant waste and improves material utilization efficiency [20]. Despite these benefits, the application of microreactors for CD synthesis still faces several challenges. Firstly, the complex fluid dynamics within microchannels can result in uneven mixing of reactants, which negatively affects reaction uniformity and efficiency. Secondly, the small channel size complicates the precise control of temperature and pressure, leading to potential instability in reaction conditions, which in turn affects product quality and consistency. Furthermore, issues related to imprecise reactant delivery, cleaning, maintenance, and technical challenges in scaling up the process for large-scale production continue to limit the broader application of microreactors. Therefore, optimizing the design and control strategies of microreactors to enhance their performance in CD synthesis remains a key focus of current research.

To overcome the limitations of traditional microreactors in CD synthesis, the combination of ultrasound and biomimetic design has emerged as a promising technological approach. The ultrasonic cavitation effect generates local high-temperature and high-pressure environments within microreactors, facilitating rapid heat transfer and intense reactions of reactants, thereby significantly enhancing both reaction rate and uniformity [[21], [22], [23]]. Additionally, the shear forces exerted by ultrasound on the solution prevent the aggregation and precipitation of reactants, ensuring greater stability in product quality [24]. On the other hand, biomimetic design provides innovative solutions for optimizing microreactor design. By mimicking natural fluid transport structures, such as tree roots and leaves [[25], [26], [27], [28]], researchers can design microchannels with superior fluid dynamics. These biomimetic structures not only improve reactant flow characteristics within the microchannels, enhancing reaction uniformity, but also increase reaction efficiency. Thus, the integration of ultrasound with biomimetic design offers a novel approach to overcoming challenges such as uneven fluid mixing and imprecise reaction control, ultimately improving CDs’ quality.

Ultrasound not only enhances mixing and mass transfer efficiency in microreactors, but also enables precise control over the spectral properties and size distribution of quantum dots (QDs). In our previous studies, we used contact ultrasound to finely tune the multicolor spectrum and cross-dimensional structures of perovskite QDs [[29], [30], [31]]. Our findings demonstrated that ultrasonic energy can induce morphological and size changes in QDs. In contrast to the room-temperature synthesis conditions typically used for perovskite QDs, the synthesis of CDs in ultrasonic microreactors occurs under the coupling of multiple physical fields, including acoustic, thermal, and pressure fields. This unique environment enhances the complexity of the synthesis process. However, the mesoscopic behaviors induced by cavitation, coupled with the limitations of current experimental measurement techniques, make it challenging to accurately elucidate the multivariate coupling mechanisms during CDs growth within confined spaces. This complexity hampers the establishment of a clear relationship between multivariate coupling and CDs morphology control, making it difficult to predict and control the growth process, and thereby complicating the preparation of CDs with controlled properties. To address this challenge, we employed computational fluid dynamics simulations and digital imaging techniques to compensate for the limitations of traditional experimental analysis. These tools enabled us to develop heat and mass transfer models, providing deeper insights into how fluid flow processes within ultrasonic microchannels influence CD properties.

In this study, we developed an ultrasonic microreactor inspired by leaf vein structures for the synthesis of indigo-emitting CDs with tunable morphology and optical properties. To optimize heat and mass transfer within the microchannel and gain a deeper understanding of fluid dynamics, we utilized COMSOL Multiphysics simulations to explore the effects of key design parameters—including leaf vein contours, fractal angles, depth-to-width ratios, and inlet configurations—on flow characteristics within the microchannels. To further enhance ultrasonic energy transfer, we directly coupled the ultrasonic transducer to the microreactor, thereby creating a high-performance ultrasonic microreactor. By refining both physical and numerical models, we identified the optimal ultrasonic frequency and power, significantly improving reactor efficiency. These findings were validated experimentally, where we observed fluid flow behavior inside the biomimetic leaf vein-inspired ultrasonic microreactor, confirming the accuracy of our simulations. Additionally, we established a robust ultrasonic microreactor system capable of synthesizing indigo-emitting CDs. Moreover, we examined the influence of multivariate coupling factors, such as flow rate, temperature, and ultrasonic power, on the formation, development, and evolution of the CDs' fluorescence spectra. This comprehensive approach seeks to provide insightful understanding of the synthesis mechanisms, thereby paving the way for optimizing the properties of CDs for various applications in materials science and nanotechnology.

## 2 Experimental sections

### 2.1 Chemicals and materials

Ammonium citrate (AR, ≥ 98.5 %), N,N-dimethylformamide (DMF, AR, ≥ 99.5 %), and deionized water (H_2_O, ≥ 18 MΩ·cm) were all purchased from Shanghai Aladdin Biochemical Technology Co., Ltd. All materials used in this study were used as received, without any further purification.

### 2.2 The structure design of leaf vein-inspired microchannels

**Fig. 1:**
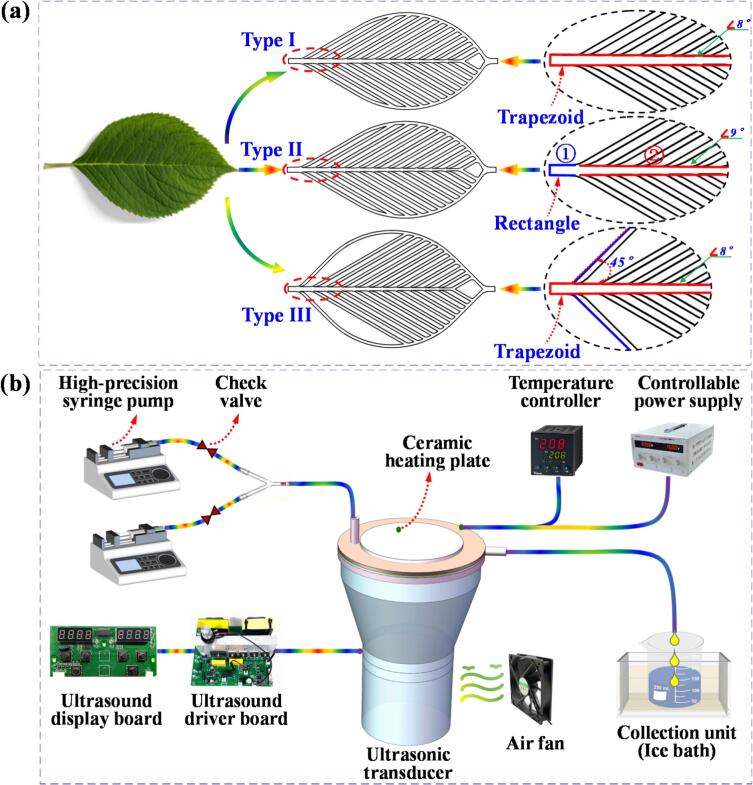
(a) Three types of leaf vein profile structures, labeled as Type I, Type II, and Type III, are presented for comparison. The primary distinction between Type I and Type II lies in the design of the main channel: in Type I, the main channel maintains a trapezoidal shape from the inlet to the twelfth branch, whereas in Type II, the main channel is rectangular (1) at the inlet and gradually transitions into a trapezoidal shape (2) from the first to the twelfth branch. The key difference between Type I and Type III is found in the outer contour angle: Type I features an outer contour angle that aligns with the fractal angles of the branching channels, whereas in Type III, the outer contour angle is fixed at 45°, regardless of the fractal angles of the branching channels. The trapezoidal main channel design in Type I and Type III enhances the uniformity of fluid flow within the microchannel, promoting smoother and more stable flow, which ultimately leads to improved overall system performance. (b) Schematic representation of the components of the biomimetic leaf vein ultrasonic microreactor system.

Nature has evolved numerous efficient network structures over millions of years, one of the most notable being the venation system of plants (Fig. 1a). These structures, characterized by their fractal patterns, have developed through natural selection to exhibit exceptional fluid dynamic properties. They optimize energy consumption, minimize resistance during fluid flow, and improve the efficiency of material and energy transfer. As a result, they offer significant advantages in reducing energy losses and enhancing transport efficiency.

Motivated by these natural systems, and recognizing the potential for manufacturing replication, we designed a microchannel based on the fractal structure of leaf veins (see Fig. 1a). This bio-inspired design aims to replicate the inherent benefits of leaf venation, particularly in terms of enhancing fluid flow uniformity. By incorporating this fractal pattern into the microchannel, we aim to reduce energy consumption and improve the overall efficiency of material and energy transfer, thereby achieving superior performance at the microscale.

### 2.3 Construction of the ultrasonic microreactor system

Fig. 1b illustrates the five main components of the leaf vein-inspired ultrasonic microreactor system: (1) Feed Delivery Unit – This unit consists of two high-precision syringe pumps, two check valves, and a Y-shaped connector. It is designed to accurately control the flow velocities and volumes of reactants, ensuring precise mixing ratios within the microreactor. (2) Ultrasonic Control Unit – This unit includes an ultrasonic transducer (SD100, Beijing Ocos Ultrasonic Technology Group Co., Ltd., with a power output range of 60–100 W and a frequency range of 20–28 kHz), a driving plate, and a display panel. It is responsible for regulating the output power, frequency, and intensity of the ultrasonic waves, ensuring efficient and uniform energy transmission throughout the reaction. (3) Microreactor Unit – The microreactor is structured with two layers: the bottom layer contains embedded microchannels, which ensure uniform and efficient fluid flow, while the top layer features sealing gaskets and a cover plate with strategically placed inlet and outlet ports. These components help control the direction and flow velocity of the fluid, facilitating effective interaction between the reactants and ultrasonic energy for enhanced reaction efficiency. (4) Temperature Control Unit – Comprising a temperature controller, an adjustable power supply, and a ceramic heating element, this unit provides precise temperature regulation from ambient up to 240 °C. It supports a wide range of reaction conditions and ensures that the temperature is maintained within the required range to optimize synthesis processes. (5) Collection Unit – This unit consists of a beaker and a cooling tank, which work together to collect and rapidly cool the reaction products. This rapid cooling process preserves the integrity and stability of the products, ensuring their suitability for further analysis and long-term storage.

### 2.4 Visual observation of fluid flow behavior in the ultrasonic microreactor

High-speed imaging was utilized to observe the dynamic fluid flow within the microchannel under ultrasonic excitation. The microreactor was positioned on a microscope stage, and the high-speed camera was calibrated to an appropriate frame rate to capture the region of interest. The syringe pump was activated, and once a steady flow was established, baseline images were recorded without the application of ultrasonic excitation. Subsequently, ultrasonic waves were introduced, and the resulting instantaneous changes in the flow field were captured. These changes included the formation and collapse of cavitation bubbles, the generation and evolution of vortices, disturbances at the fluid interface, and variations in the velocity distribution.

A comparative analysis of the images taken before and after the application of ultrasonic excitation allowed for a detailed examination of the flow dynamics. This approach provided both visual and quantitative evidence, offering valuable insights into the evolution of the flow characteristics under ultrasonic influence and supporting a deeper understanding of the underlying mechanisms driving fluid behavior in the microreactor.

### 2.5 Synthesis of luminescent CDs using the ultrasonic microreactor

A creative method for synthesizing luminescent CDs was developed using the biomimetic leaf vein ultrasonic microreactor. The synthesis involved anhydrous ammonium citrate (precursor), DMF (solvent and modifying agent), and deionized water (solvent). Ammonium citrate was dissolved in deionized water to prepare precursor solution A, which was then mixed with an equal volume of DMF to form precursor solution B.

Before injection, the temperature control system was activated to heat the microreactor to the desired temperature. The ultrasonic frequency and power were optimized to achieve stable cavitation intensity. The microchannels were thoroughly flushed using syringe pumps to ensure consistent reaction conditions. During the synthesis process, solutions A and B were continuously delivered at a constant flow velocity. The temperature control system maintained thermal stability, ensuring effective coupling of ultrasonic cavitation with the high-temperature, high-pressure environment inside the reactor. This synergy facilitated the rapid condensation and carbonization of the precursors, resulting in CDs with high fluorescence efficiency. After the reaction, the products were collected in a beaker and rapidly cooled in a cooling tank to preserve their purity and stability. This method provided enhanced control over the synthesis process, yielding high-quality luminescent CDs. In this study, the flow velocity of the precursor solutions was set between 0.2 and 0.8 mL/min, with the reaction temperature ranging from 170 to 210 °C. The precursor concentration was maintained at 0.2 mol/L, the ultrasonic frequency was set between 20 and 28 kHz, and the ultrasonic power ranged from 60 to 100 W.

### 2.6 Characterization

The structural design and optimization of the microchannels were performed using COMSOL Multiphysics 6.2 (COMSOL, Sweden). Morphology and size distribution were characterized by transmission electron microscopy (TEM, FEI Talos F200x, USA) at an accelerating voltage of 200 kV. TEM sample preparation for the CDs involved ultrasonication for dispersion, drop casting, drying, and cleaning. UV–vis absorption spectra were recorded using a spectrophotometer (TU-1901, PuXi, China), with an excitation wavelength of 360 nm and a spectral range of 300–800 nm. Photoluminescence (PL) spectra were measured with a fluorescence spectrophotometer (RF-6000, Shimadzu, Japan), using an excitation wavelength range of 360–480 nm and an emission range of 400–700 nm. Fluorescence lifetime measurements and Photoluminescence quantum yield (PLQY) were conducted using a steady-state and transient fluorescence spectrometer (FLS1000, Edinburgh Instruments, UK), with an excitation wavelength of 365 nm.

To assess the flow uniformity within the designed microchannel, we employed the following method: velocity measurements were conducted at key positions along each branching channel, specifically at z = *h*/2, y = 0, and at points corresponding to half the lateral width (*D*/2) of the branching channels. The flow velocity at the symmetric branch positions was calculated, and the average velocity of each symmetric branch channel was derived. To quantify flow uniformity, the flow velocity discrepancy rate within the microchannel was used as an evaluation criterion. The formula for calculating the velocity discrepancy rate (*δ*) is as follows [25]:

(1) $$\delta =\frac{\sum_{i=1}^{n}\frac{|V_{1}\left(i\right)-V_{2}\left(i\right)|}{V_{2}\left(i\right)}\times 100\%}{n}$$

With the main channel as the dividing line, the symmetrical branching channels are split into upper and lower sections. *V*_1_(i) represents the average flow velocity of the branching channels in the upper cross-sectional sampling point, while *V*_2_(i) represents the average flow velocity of the branching channels in the lower cross-sectional sampling point. *n* is the total number of branching channels.

The PL decay lifetime was measured using an Edinburgh Instruments FLS980 spectrometer (Edinburgh Instruments, UK). The PL decay curves were fitted with multi-exponential functions as described by the following expression [32]:

(2) $$A\left(t\right)=\sum_{i=1}^{n}A_{i}\exp\left(\frac{-t}{\tau_{i}}\right)$$

where *A(t)* is the PL intensity at time *t*; *A_i_* denotes the relative amplitude of each lifetime component at *t* = 0; and *τ_i_* is the corresponding decay time. The average lifetime *τ_avg_*. was calculated using the following equation [33]:

(3) $$\tau_{\mathrm{avg}.}=\frac{A_{1}\tau_{1}^{2}+A_{2}\tau_{2}^{2}}{A_{1}\tau_{1}+A_{2}\tau_{2}}$$

## 3 Results and discussion

### 3.1 Numerical simulation and optimization of flow field structure in leaf vein-inspired microchannels

The performance of CDs is influenced not only by reaction parameters but also by the functional structure of the microreactor. Previous studies have demonstrated that the microchannel design plays a critical role in determining both the quality and functionality of CDs [17,25,34,35]. Optimizing the microchannel structure enables more uniform flow velocities and efficient heat transfer, which are essential for enhancing the condensation and carbonization processes involved in CD synthesis. Therefore, the design and optimization of microchannels are critical for producing high-performance CDs.

This section introduces a physics-informed simulation–optimization framework aimed at improving the flow field structure of microchannels through velocity and pressure distribution data. The goal is to adjust the geometric parameters of the microchannel to enhance flow uniformity and overall system performance. Detailed numerical simulations were performed using COMSOL Multiphysics to analyze fluid flow characteristics within the leaf vein-inspired microchannels. The study focused on the effects of various geometric factors, such as the biomimetic leaf vein profile structure, fractal angle (*β*), depth-to-width (h/D) ratio, and different inlet configurations. Optimizing these parameters results in more uniform fluid flow, leading to significant improvements in heat and mass transfer efficiency within the system.

Before the simulation, we calculated the key dimensionless numbers based on the physical properties of the experimental substances and the microchannel structural parameters. These numbers defined the simulation boundary conditions. Using the channel dimensions and fluid properties approximated to those of the aqueous precursor solution, we determined the relevant dimensionless numbers to characterize the hydrodynamic regime during synthesis. The Reynolds number (*R*_e_) is consistently in the laminar range, with values spanning approximately 1.99 to 13.7, confirming that the flow remains predominantly laminar throughout the studied conditions. Correspondingly, the Peclet number (*P*_e_) varies from approximately 9.6 × 10^4^ to 1.16 × 10⁶, indicating that convection significantly dominates mass transport processes over molecular diffusion. The Capillary number (*C*_a_), ranging from ∼6.8 × 10^−5^ to 2.7 × 10^−4^, along with Weber numbers (all cases showing *W_e_* ≪ 1), reinforces the conclusion that bubble stability is primarily governed by surface tension effects, with negligible inertial breakup occurring under steady flow conditions. In contrast, under ultrasound conditions, the streaming Reynolds number (*R*_es_) ranges from approximately 1 to 103, signaling the onset of substantial acoustic streaming effects, which enhance mixing and cavitation processes. The Strouhal number (*S_t_*) is significantly greater than 1, further indicating dynamic interactions between the flowing fluid and the ultrasonic waves. The detailed calculations, assumptions, and additional context for these dimensionless numbers can be found in the Supplementary Information (SI 1).

#### 3.1.1 Effect of leaf vein profile structure on fluid dynamics in biomimetic microchannels

**Fig. 2:**
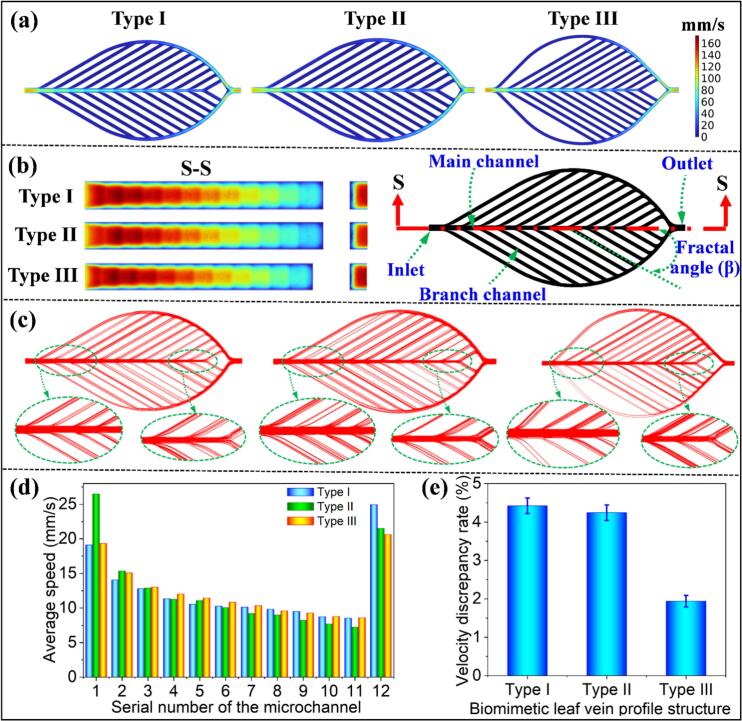
(a) Velocity distribution contour maps of the microchannel flow for different leaf vein profile structures in the XOY plane. (b) Velocity distribution contour maps of the microchannel flow for different leaf vein profile structures in the ZOX plane (S-S section view). (c) Streamline distribution contour maps of the microchannel flow for different leaf vein profile structures in the XOY plane. (d) The effect of different leaf vein profile structures on the flow velocity distribution within each branching channel. (e) *δ* values for Type I, II, and III microreactors, respectively.

This study first investigates the influence of different leaf vein profile structures on the fluid characteristics within the biomimetic leaf vein microchannels, focusing specifically on how these structures influence flow velocity distribution. The flow velocity distribution was analyzed in both the XOY plane (Fig. S1a) and the ZOX plane (Fig. S1b) of the microchannel, with the results visualized through velocity contour maps (Fig. 2, Fig. 2).

To begin, we analyzed the velocity distribution within the main and branch channels in the XOY plane. Under constant inlet velocity conditions, all three microchannel profiles—Type I, Type II, and Type III—showed similar velocity distributions. Higher velocities were observed near the inlet, outlet, and main channel, while relatively lower velocities were present within the branch channels (Fig. 2a). This indicates a relatively uniform flow pattern across the different designs, with the main flow path (i.e., the inlet and outlet regions) experiencing the highest velocities, and the branching regions experiencing slower flow due to increased resistance.

To further investigate the velocity distribution, the branch channels were divided into 12 groups based on their distance from the inlet. The branch closest to the inlet was designated as Group (1), and the branch closest to the outlet was designated as Group 12 (see Fig. S1c). Symmetrical branches were grouped together for comparative analysis. The velocity distribution contour maps shown in Fig. 2a indicate that symmetrical branches within the same group exhibited similar velocities for Type I, II, and III, respectively. As the group number increased (from Group (1) to Group 12), the velocity within the branch channels gradually decreased (e.g. Type I). This reduction in velocity is attributed to the progressive redistribution of fluid from the main channel to the branch channels, which leads to a corresponding decrease in both velocity and pressure within the main channel (see Fig. S2).

In Fig. 2b, it is observed that the velocity initially decreases and then increases as the flow moves from the inlet toward the outlet (S-S section view), with a characteristic step-like variation in the velocity contours. This behavior results from the splitting of the flow at each branch, which causes the flow to decelerate before accelerating again as it reenters the main channel. Such flow dynamics are critical for understanding the distribution of reactants and the effectiveness of the reaction process within the microchannels. Additionally, in the ZOX plane, where the flow is analyzed along the vertical depth direction, the velocity distribution is not uniform at the same horizontal position. Notably, at the interface between the channel surface and the bottom, significant velocity gradients are observed. This phenomenon is attributed to the fluid properties, including viscosity and density variations, as well as the interaction forces between the fluid and the microchannel surface [36]. These effects are particularly pronounced near the walls, where the flow is influenced by the no-slip condition, leading to a decrease in velocity close to the surface compared to microchannel centerline.

Further analysis of the streamline distribution in the XOY plane (see Fig. 2c) revealed that, regardless of the profile structure (Type I, II, and III), no significant vortex formation occurred at the junction between the main and branch channels. This observation suggests that the flow remains relatively uniform, which is beneficial for reducing energy consumption and minimizing flow resistance. Notably, the streamline distribution in Type III was found to be more uniform and smoother than in Type I and Type II, indicating its superior potential for optimizing fluid flow. This enhanced flow uniformity is crucial for improving fluid transport efficiency and reducing localized pressure losses, which are essential factors in the optimization of microfluidic system designs.

To quantitatively assess the effect of different profile structures on flow uniformity, we introduced the *δ* (see Equation (1)) as a key metric. It is worth noting that a lower *δ* indicates a more uniform velocity distribution across the branch channels, contributing to improve the uniformity of heat and mass transfer. Fig. 2d shows that the average velocity of the branch channels first decreases and then increases as the group number increases, suggesting a shift in flow dynamics along the microchannel. Fig. 2e demonstrates the significant effect of different profile structures on the *δ*. Both Type I and Type II exhibited similar discrepancy rates, ranging from 4.0 % to 4.5 %, while Type III achieved the lowest rate at 1.942 %. This result underscores Type III’s superior performance in maintaining a more uniform flow distribution, making it the most optimal structure among the three.

#### 3.1.2 Effect of fractal angle on fluid dynamics in biomimetic microchannels

Numerical simulations were conducted on both the XOY and ZOX planes to elucidate the influence of fractal angle (*β*) on the flow uniformity within biomimetic leaf vein microchannels. Velocity contour plots (Table 1) were employed to visualize and analyze the velocity distribution and assess the impact of different fractal angles on flow uniformity.

**Table 1:** (a) Velocity distribution contour maps of the main and branching channels for different vein profile structures at fractal angles of 30°, 45°, and 60° in the XOY plane. (b) Velocity distribution of the main channel in the microreactor for different vein profile structures and fractal angles in the ZOX plane.

Under the conditions of identical vein contour geometry and constant inlet velocity, microchannels with three fractal angles (30°, 45°, and 60°) were investigated. The results indicate that the overall velocity distribution patterns are similar across the different angles: higher velocities are observed at the inlet and outlet regions, while velocities within the branch channels are relatively lower (Table 1a). At the branch junctions, the fluid velocity progressively decreases as the flow moves into the branches.

A detailed analysis of the velocity contours on the XOY plane further shows that the velocity within the branches decreases from group (1) to group 12. This trend arises from the gradual diversion of flow from the main channel into the branches, which leads to a corresponding reduction in both velocity and pressure along the main channel. The concentrated flow at the inlet results in higher velocity, whereas downstream, the combined effects of flow redistribution, friction losses, and increased flow resistance contribute to a further decline in velocity within the branch channels [37,38]. Moreover, the trend of velocity reduction in the branches becomes more pronounced as the fractal angle increases, indicating that larger fractal angles enhance the flow redistribution effect and flow resistance, thereby significantly reducing velocity. The increased flow resistance associated with larger fractal angles further disrupts the flow uniformity, which could potentially impact the performance of microfluidic systems. Therefore, the optimization of fractal angles plays a crucial role in balancing flow distribution and minimizing resistance in such systems.

The velocity distribution on the ZOX plane (Table 1b) shows that for all fractal angles, the main channel velocity decreases gradually from the inlet to the outlet, with a sharp rise in velocity near the outlet. For the Type I microchannel, the 30° fractal angle yields the most uniform velocity distribution, while the 60° fractal angle results in the greatest velocity fluctuations. In the Type II microchannel, velocity distribution is relatively uniform at both 30° and 45°, particularly in the central region of the channel, reflecting more favorable flow characteristics. The Type III microchannel exhibits consistently high velocity uniformity across all angles, with the most stable velocity distribution observed at 30°. These findings further validate the advantage of the Type III microchannel in optimizing velocity distribution. Specifically, the Type III channel at a 30° fractal angle not only effectively suppresses velocity fluctuations but also enhances overall flow uniformity and stability, demonstrating significant potential for applications in fluid transport systems.

**Fig. 3:**
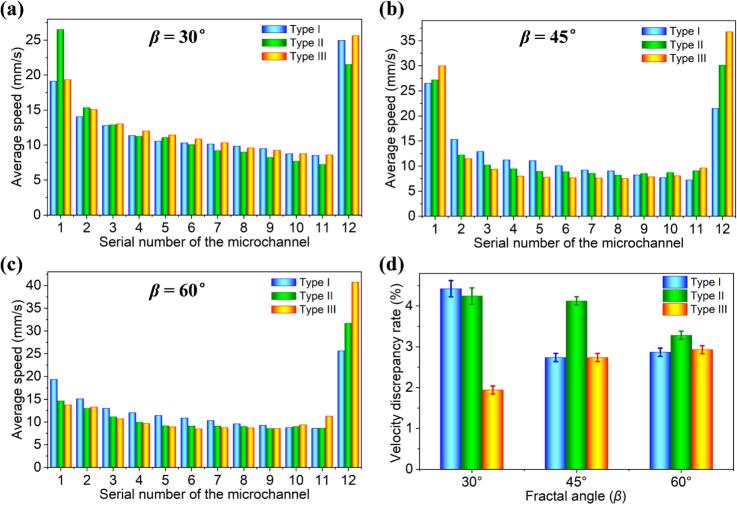
Average flow velocity distribution in the branching channels of the microreactor for different vein profile structures at various fractal angles in the XOY plane: (a) *β* = 30°, (b) *β* = 45°, (c) *β* = 60°. (d) *δ* values of the Type I, II, and III microreactors at different fractal angles, respectively.

To gain a more comprehensive and in-depth understanding of how different fractal angles affect the uniformity of fluid flow in microchannels, we thoroughly analyzed the flow velocity distribution in each branch channel and calculated the *δ* across the entire flow field. By examining Fig. 3a-c, we observed an intriguing phenomenon: the average flow velocity in the branch channels first decreases and then increases. This behavior not only reveals the complex distribution and transfer processes of fluid within the microchannel network, but also offers a new perspective for understanding the flow characteristics of microchannels. At the same time, this trend clearly demonstrates the significant impact of different fractal angles on the *δ* in the branch channels.

Fig. 3d presents the *δ* values for Type I, II, and III microreactors at various fractal angles. At a fractal angle of 30°, the *δ* values are recorded as follows: 4.42 % for Type I, 4.24 % for Type II, and 1.94 % for Type III microreactors. When the fractal angle is adjusted to 45°, the *δ* values change to 2.74 % for Type I, 4.13 % for Type II, and 2.74 % for Type III. At a fractal angle of 60°, the *δ* values observed are 2.88 % for Type I, 3.28 % for Type II, and 2.93 % for Type III. Notably, the Type III microchannels consistently exhibit the lowest *δ* value (∼1.94 %) across all tested fractal angles, confirming their superior fluid flow uniformity. This finding supports the conclusion that microchannels with a 30° fractal angle enhance flow performance, indicating the potential for optimizing microreactor designs to improve efficiency in various applications.

Under constant inlet injection velocity, Fig. 3d shows minimal deviations in flow velocity among the three fractal angles (30°, 45°, and 60°), further validating that the fractal network structure enhances fluid velocity uniformity. The unique branching characteristics and self-similarity of the fractal network effectively disperse and balance fluid flow, ensuring consistent flow velocity across different branch channels. In particular, the 30° fractal angle microchannel in Type III demonstrates the smallest *δ*, emphasizing its advantage in maintaining flow uniformity and greater efficiency in fluid distribution and transport, critical for microfluidic systems that require precise flow control.

#### 3.1.3 Effect of the depth-to-width ratio on fluid dynamics in biomimetic microchannels

We investigated the effect of the h/D ratio on flow uniformity in microchannels by analyzing five sets of microchannels with varying h/D ratio. Velocity contour maps in the XOY plane were obtained for each set (as shown in Table 2). These results provide clear, intuitive evidence to better understand how the h/D ratio influences the fluid flow characteristics within microchannels.

**Table 2:** Velocity distribution contour maps of the main and branching channels within the Type III microreactor at different h/D ratios and fractal angles in the XOY plane.

When examining the velocity distribution in microchannels with the same fractal angle but varying h/D ratio, the velocity contours in the XOY plane show that the velocity distribution remains symmetric as the h/D ratio changes. This observation further confirms the superiority of the biomimetic venation model in promoting flow uniformity. We also analyzed the velocity distribution in microchannels with different fractal angles but identical h/D ratio. The experimental results indicated that, for the same h/D ratio, microchannels with a 30° fractal angle exhibited higher average velocities, while those with a 60° fractal angle showed relatively lower average velocities. Among the three fractal angles, microchannels with an h/D ratio of 3.0 displayed the best average velocity across all five h/D ratio sets, demonstrating ideal fluid distribution characteristics. The pressure contour maps in Table S2 further suggest that optimizing the h/D ratio can effectively improve velocity distribution uniformity, thus enhancing the heat exchange efficiency.

Further analysis of the velocity streamline distribution in the XOY plane (shown in Table S1) revealed that increasing the h/D ratio did not lead to significant vortex formation at the junctions between the main and branch channels. This indicates that a higher h/D ratio substantially optimizes flow uniformity. As the h/D ratio increases, the streamline distribution becomes more uniform, primarily because the larger h/D ratio facilitates better fluid dispersion within the microchannel. Notably, in microchannels with a 30° fractal angle, the streamline distribution was particularly uniform, and the fluid flow was smoother. In contrast, microchannels with a 45° fractal angle, while displaying relatively smooth flow lines, showed slightly less uniformity. Under the same h/D ratio, smaller fractal angles (30°) are more conducive to achieving uniform streamline distribution.

Additionally, we examined the velocity distribution of the main channel under different fractal angles and aspect ratios in the ZOX plane, as shown in Fig. S3. For the 30° fractal angle microchannels, regardless of the h/D ratio, the velocity distribution was relatively uniform with smooth variations, indicating smaller velocity fluctuations and greater flow stability within the microchannel. In comparison, microchannels with a 45° fractal angle showed noticeable velocity non-uniformity at the same h/D ratio, with more significant fluctuations. This suggests that larger fractal angles may exacerbate velocity non-uniformity, leading to greater fluid flow instability. Microchannels with a 60° fractal angle exhibited the highest velocity non-uniformity under all h/D ratio conditions, primarily due to the increased turning angle of the fluid, which alters the fluid dynamics and amplifies velocity fluctuations. However, as the h/D ratio increases, the velocity distribution for all fractal angles gradually becomes more uniform, demonstrating that a larger h/D ratio promotes fluid dispersion within the microchannel, thereby reducing local velocity variations and improving flow uniformity and stability.

**Fig. 4:**
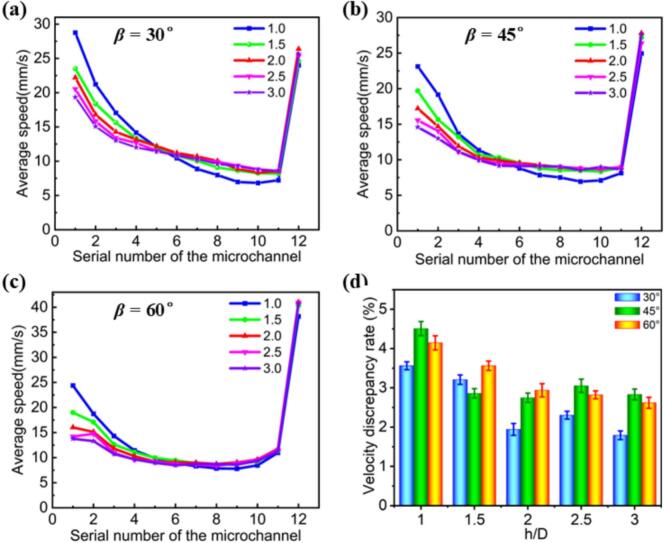
Average flow velocity distribution in the branching channels of the Type III microreactor for h/D ratios at various fractal angles in the XOY plane: (a) *β* = 30°, (b) *β* = 45°, (c) *β* = 60°. (d) *δ* values of the Type III microreactor for different h/D ratios at various fractal angles.

To further investigate the velocity distribution and flow uniformity of microchannels with different h/D ratios, we also analyze variations in the average flow velocity of each branch channel and the flow velocity disparity across the entire microchannel, as shown in Fig. 4. From Fig. 4a-c, we observe that, for a constant fractal angle (e.g., 30°), the average flow velocity of each branch channel follows a similar trend. As the h/D ratio increases (i.e., 1.0–3.0), the average flow velocity in the branch channels gradually decreases. This phenomenon can be attributed to the flow characteristics in microchannels: as the fluid enters the channel, it initially disperses and then gradually converges, causing the flow velocity to first decrease and later increase.

Further analysis reveals that, for microchannels with the same h/D ratio, the average flow velocity of each branch channel does not change monotonically but exhibits a trend of first decreasing and then increasing. This behavior is closely related to the fluid flow state within the microchannel. Initially, fluid dispersion causes a decrease in flow velocity; however, as the channel geometry changes, the fluid gradually converges, leading to a recovery in flow velocity. Therefore, the fluctuation in flow velocity reflects the characteristic behavior of the fluid flow, where it first disperses and then converges.

Additionally, we analyzed the flow velocity disparity across all branch channels of microchannels with different fractal angles and h/D ratio, as shown in Fig. 4d. The results demonstrate that among the five data sets analyzed, the *δ* values reach relative maxima when the h/D ratio is 1, with measurements of 3.56 % for *β* = 30°, 4.51 % for *β* = 45°, and 4.15 % for *β* = 60°. Conversely, at an h/D ratio of 3, the *δ* values showcase relative minima, recorded at 1.79 % for *β* = 30°, 2.83 % for *β* = 45°, and 2.62 % for *β* = 60°, with *β* = 30° exhibiting the lowest value. Furthermore, as the h/D ratio transitions from 1 to 3, the average *δ* values for *β* = 30°, 45°, and 60° are calculated to be 2.56 %, 3.20 %, and 3.22 %, respectively. This analysis reiterates that the configuration with *β* = 30° consistently shows the minimum *δ* value. Based on these findings, we conclude that the microchannel with a 30° fractal angle and an h/D ratio of 3.0 exhibits optimal flow uniformity.

#### 3.1.4 Effect of inlet configuration on fluid dynamics in biomimetic microchannels

To examine the impact of inlet configuration on flow uniformity, we conducted a comprehensive analysis of dual-inlet microchannels, generating velocity contour maps in both the XOY and ZOX planes, as shown in Table 3.

**Table 3:** Velocity distribution contour maps of the internal channels within the Type III microreactor with double- and single-inlet at various h/D ratios in the XOY plane, and velocity distribution contour maps of the main channels within the Type III microreactor with double- and single-inlet in the ZOX plane.

The study of microchannel flow characteristics revealed that, under a constant inlet injection velocity, the flow distribution in double-inlet microchannels followed trends similar to those observed in single-inlet microchannels. Specifically, both types exhibited higher flow velocities near the inlets and outlets, with lower velocities in the branching channels. Notably, the flow velocities at the inlet and outlet regions of double-inlet microchannels were generally higher than those in single-inlet microchannels. This difference can be attributed to the inlet effect in fluid dynamics, where boundary conditions lead to an increase in flow velocity at the inlets.

Further analysis of the streamline distribution (Table S3) showed no significant vortex formation at the junctions between the main and branch channels, either in double-inlet or single-inlet microchannels. This indicates an ideal streamline distribution, with no flow separation or vortex formation, suggesting stable flow behavior. These findings are crucial for the design and optimization of microfluidic devices. Additionally, we identified that the Type III microchannel structure, with a fractal angle of 30°, exhibited unique advantages in optimizing fluid flow. This structural design effectively reduces flow resistance, enhances flow efficiency, and improves the thermal and mass transfer performance of microfluidic devices.

**Fig. 5:**
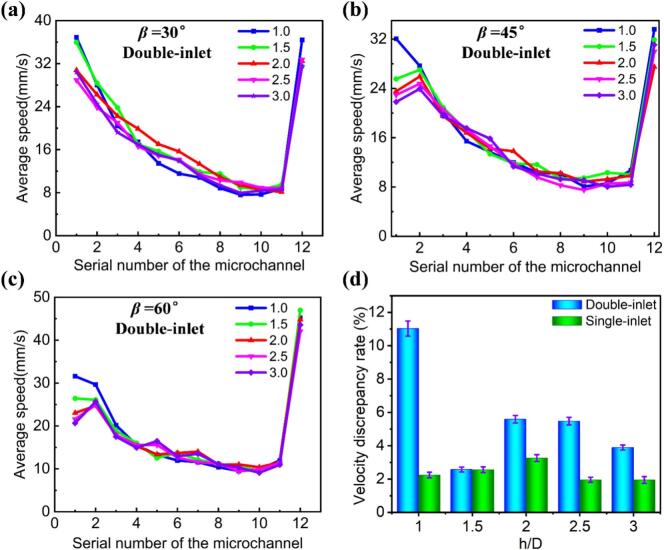
Average velocity distribution in the branching channels of the double-inlet Type III microreactor at different h/D ratios and fractal angles in the XOY plane: (a) *β* = 30°, (b) *β* = 45°, (c) *β* = 60°; (d) *δ* values of the Type III microreactor at different fractal angles and h/D ratios.

To further assess the velocity distribution and flow uniformity in double-inlet microchannels at varying h/D ratio, we analyzed the average flow velocity of the branch microchannels and the velocity disparity across the entire microchannel, as shown in Fig. 5. The results indicate that when the *β* is set to 30°, the average flow velocity exhibits a consistent trend of first decreasing and then increasing as the number of channels varies from the first to the twelfth group, regardless of changes in the h/D ratio. The measured average velocities range from 7.6 to 36.9 mm/s. In contrast, for *β* values of 45° and 60°, the trends in average flow velocity vary with different h/D ratios. Specifically, at an h/D ratio of 1, both β configurations show a pattern of initially decreasing and then increasing average velocities. However, when the h/D ratio is between 1.5 and 3, the average velocities first increase, then decrease, and subsequently increase again.

Additionally, we analyzed the *δ* values of all branch channels under varying inlet conditions and h/D ratios, as depicted in Fig. 5d. The *δ* values in the double-inlet microchannels were consistently higher than those in the single-inlet microchannels across all h/D ratios, with values of 11.03 %, 2.57 %, 5.59 %, 5.47 %, and 3.89 % for h/D = 1.0, 1.5, 2.0, 2.5, and 3.0, respectively. Notably, at h/D ratios of 1.0 and 2.5, the *δ* in the double-inlet microchannels was significantly higher than that in the single-inlet microchannels, highlighting the clear advantage of the single-inlet design in enhancing the uniformity of velocity distribution.

In summary, after optimizing the aforementioned parameters, the fundamental profile and key characteristics of the microchannel have been established. The specific dimensions and geometry are presented in Fig. S4, with comprehensive details provided in the supplementary materials.

### 3.2 Structural design of the biomimetic leaf vein ultrasonic microreactor

To enhance ultrasonic energy transmission efficiency, we directly coupled the ultrasonic transducer with the microreactor, forming a high-efficiency ultrasonic microreactor. By carefully optimizing the structural parameters, the system was maintained in a resonant state along the longitudinal axis, ensuring that the microreactor was positioned at the vibration mode’s antinode. This positioning maximized the displacement response and facilitated a uniform acoustic field distribution.

Given that resonance behavior is highly sensitive to the size, geometry, and material properties of each component, traditional numerical methods alone are insufficient for determining the complete set of design parameters. To address this challenge, we incorporated findings from previous research, as well as relevant design knowledge and empirical experience, to predefine several critical structural parameters (refer to Figs. S5, S6, and S7). Subsequently, finite element simulations were conducted to evaluate the acoustic performance of the microreactor under operational conditions. Particular attention was paid to the uniformity of the acoustic field and the efficiency of energy transmission. These simulations provided theoretical support for structural optimization, ensuring effective and stable ultrasonic energy delivery within the microreactor.

#### 3.2.1 Design and structural optimization of the biomimetic ultrasonic microreactor

The ultrasonic microreactor consists of three primary components: a biomimetic leaf vein microchannel plate, a transparent sealing gasket, and an upper cover plate (Figs. S5 and Fig. S6). The sealing gasket, positioned between the upper cover and the microchannel plate, creates a tightly integrated structure that prevents reagent leakage, enhances system sealing, and improves the reactor's structural stability and operational reliability. This layered assembly optimizes component interfacing, ensuring sustained high performance over extended operational periods.

To investigate the influence of ultrasonic frequency and power on flow uniformity within the microchannels, acoustic field simulations were performed. The simulation incorporated specific boundary conditions and coupling steps. In the electrostatics domain, the piezoelectric actuator was modeled with two electrical terminals: Terminal 1, constrained to 0 V, and Terminal 2, driven by a sinusoidal excitation of 220 V. This configuration generates the required electric field across the piezoelectric element, inducing mechanical vibrations via the converse piezoelectric effect. In the solid mechanics domain, a fixed constraint was applied to the bottom surface of the transducer to prevent rigid-body motion, ensuring deformation resulted solely from the piezoelectric excitation. In the pressure acoustics domain, the microchannel's inlet and outlet were modeled as hard-wall acoustic boundaries, enforcing zero normal velocity and preventing energy leakage. A structured hexahedral mesh was used for both the solid mechanics and acoustics domains. This approach allowed for accurate modeling of standing wave formation and pressure distribution within the microchannel. The coupled simulation, implemented within a Multiphysics framework, simultaneously solved the electrical, mechanical, and acoustic fields to ensure consistent energy transfer. The resulting sound pressure level (SPL) distribution maps in the XOY plane revealed significant variations in the internal branch channels of the Type III microreactor under different ultrasonic conditions (Table 4).

**Table 4:** Sound pressure level distribution contour maps of the main and branching channels within the Type III microreactor at different ultrasonic power and frequency levels in the XOY plane.

During the analysis of SPL distributions in the branch channels of a Type III microreactor under varying ultrasonic frequencies and power levels, we evaluated the acoustic field characteristics using SPL contour maps. The results demonstrate a significant variation in SPL distribution with frequency, while power is kept constant. At 20 kHz, the SPL distribution is relatively dispersed, indicating considerable attenuation and scattering of acoustic waves during propagation within the microreactor. As the frequency increases to 21 kHz, the SPL distribution becomes more uniform, promoting more homogeneous chemical reactions inside the microreactor. However, further increases to 25 kHz and 28 kHz result in non-uniform SPL patterns, likely due to suboptimal resonance effects at these frequencies.

At a fixed frequency, the overall SPL intensity increases with ultrasonic power, reflecting enhanced acoustic wave propagation strength. Among all the frequency–power combinations tested, the 21 kHz and 100 W condition exhibited the most favorable performance (see Table 4). Under this setting, the SPL contour demonstrated broad coverage and high acoustic intensity, significantly improving mixing efficiency and reaction rate within the microreactor. Additionally, the uniformity of the acoustic field suggests efficient energy utilization and minimized energy loss.

**Fig. 6:**
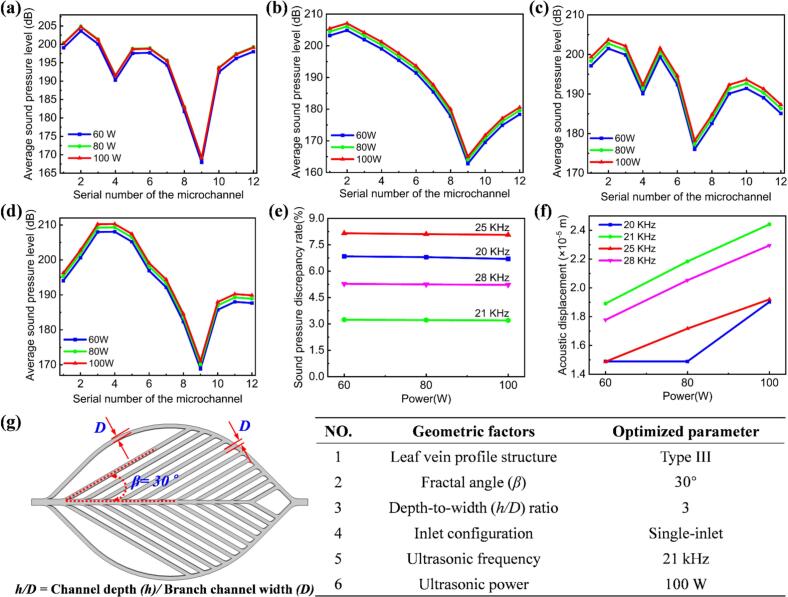
Average SPL distribution of the branching channels within the Type III microreactor at various ultrasonic frequencies and power levels in the XOY plane: (a) 20 kHz, (b) 21 kHz, (c) 25 kHz, (d) 28 kHz. (e) Sound pressure discrepancy rate of the average SPL within the microchannel as a function of ultrasonic frequency and power. (f) Sound displacement amplitude of the Type III microchannel under different ultrasonic frequencies and power levels. (g) Simplified schematic of the microchannel and optimized key structural parameters of the microchannel.

To comprehensively evaluate the impact of ultrasonic frequency and power on the uniformity of sound pressure within the microchannels, we systematically analyzed the sound pressure distribution across each branch channel and calculated the sound pressure deviation rate, as shown in Fig. 6. The results reveal considerable variation in SPLs across individual branch channels at frequencies of 20 kHz (Fig. 6a), 25 kHz (Fig. 6c), and 28 kHz (Fig. 6d). Additionally, as the number of microchannels increases, sound pressure fluctuations become more pronounced at these frequencies, indicating an unstable acoustic field. Such instability may lead to non-uniform mixing and consequently reduce reaction efficiency. In contrast, the acoustic behavior at 21 kHz (Fig. 6b) demonstrates more favorable characteristics, where the SPLs increase steadily and consistently with the number of channels. This trend suggests that, at 21 kHz and 100 W, an increase in the number of channels enhances the delivery of acoustic energy, thereby improving both fluid mixing and reaction efficiency within the microreactor. The uniformity of sound pressure distribution at this frequency is critical for ensuring effective interactions between the fluid and ultrasonic waves, facilitating better mixing and more efficient chemical reactions.

Moreover, we conducted precise characterization of the admittance properties of the ultrasonic microreactor using an impedance analyzer. Figs. S8a-b present the admittance and impedance curves of the ultrasonic microreactor, respectively. Experimental results reveal a distinct resonance peak within the frequency range of 20–28 kHz, with the resonance frequency precisely identified at 21.5 kHz. This value closely matches the numerical simulation result of 21 kHz, confirming the accuracy of our simulations.

Further analysis reveals that operating at 21 kHz and 100 W produces the maximum acoustic displacement, as shown in Fig. 6f, indicating a more pronounced cavitation effect. This enhanced cavitation intensifies chemical reaction efficiency by generating significant localized energy dissipation. Additionally, sound pressure discrepancy rate calculations show that as ultrasonic power increases, Sound pressure deviations for all four ultrasonic frequencies decrease. At 100 W, the sound pressure discrepancy rates for the four frequencies are 6.69 %, 3.20 %, 8.06 %, and 5.22 %, respectively, as shown in Fig. 6e. These results suggest that the optimal configuration for achieving both stable acoustic fields and enhanced cavitation intensity is 21 kHz at 100 W. This combination is ideal for promoting efficient and consistent chemical reactions in microreactor systems, making it highly suitable for a wide range of ultrasonic sonochemistry applications.

It is important to note that our current conclusions—identifying 21 kHz and 100 W as the optimal conditions—are based on correlation-based observations. The lack of rigorous decoupling of thermal and hydrodynamic factors during the initial optimization limits our ability to establish definitive causal relationships. To address this limitation in our future work, we will systematically vary both frequency and power while keeping flow rate and temperature constant. This approach will be complemented by localized acoustic field mapping, which will include measurements of sound pressure levels and displacement.

Given the equivalent influence of parameters such as leaf vein profile structure, *β*, h/D, and inlet configuration on fluid flow velocity, each factor was assigned an equal weight of 1. To identify the optimal parameter combination, we conducted detailed numerical simulations to analyze the impact of these factors on fluid behavior within biomimetic leaf vein microchannels. In addition, we calculated the average velocity discrepancy for each parameter. We also investigated the acoustic performance of the ultrasonic microreactor through simulations, examining how these parameters affect acoustic field uniformity and energy transfer efficiency. By comparing performance data across various parameter combinations, we identified the optimal set of parameters, corresponding to the lowest average velocity and sound pressure discrepancies. This indicates a more uniform fluid distribution and maximized acoustic field efficiency. The final optimization results are summarized in Fig. 6g, providing a clear demonstration of the overall improvement in both fluid dynamics and acoustic performance.

### 3.3 Visualization of fluid flow characteristics in biomimetic leaf vein microchannels

**Fig. 7:**
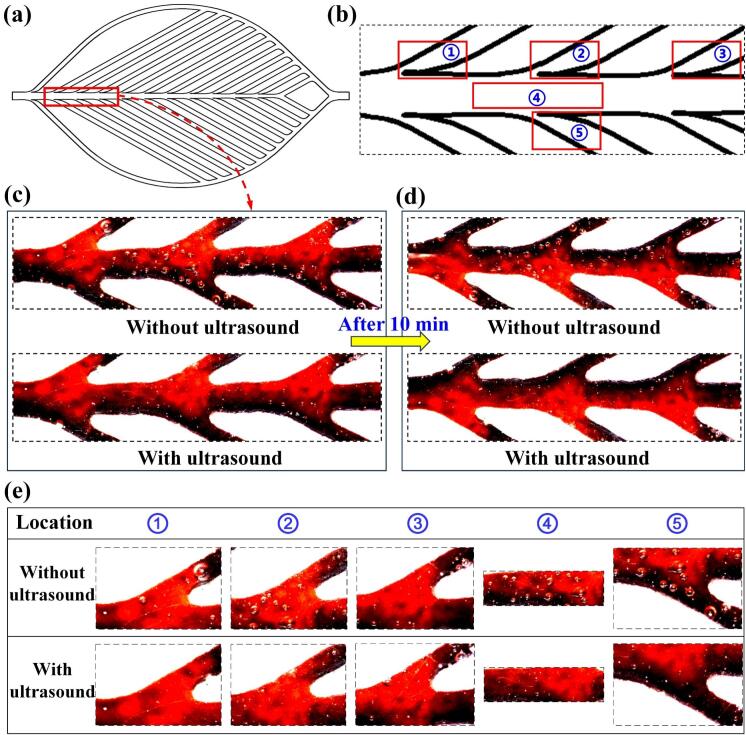
(a) Schematic of the Type III microchannel model (*β* = 30°, h/D = 3, single inlet) used to investigate fluid flow behavior. Observation points were chosen at the junctions between the main channel and the second-, third-, and fourth-level branches to assess flow characteristics both before and after ultrasonic excitation. (b) Magnified view of the selected regions for localized analysis. (c, d) Time-resolved comparison of flow patterns at identical locations, highlighting changes in bubble distribution and flow uniformity before and after ultrasound application. (e) Detailed analysis of fluid flow behavior in the regions marked in (b), focusing on the junctions of branch channels and the main trunk, with a comparison of bubble dynamics before and after ultrasonic treatment.

To investigate the influence of ultrasound on fluid dynamics within microchannels, flow visualization experiments were conducted using a Type III single-inlet microchannel model. This model featured a fractal angle of 30° and an h/D ratio of 3. Observation points were strategically selected at the junctions between the main channel and the second-, third-, and fourth-Group branches, as illustrated in Fig. 7a. Flow patterns were recorded both before and after the application of ultrasound to assess its effect on bubble behavior, flow uniformity, and mixing performance.

Comparative analysis of sequential images captured at identical locations revealed distinct differences between the non-ultrasonic and ultrasonic conditions. In the absence of ultrasound (Fig. 7c), bubbles within the fluid exhibited non-uniform spatial distribution, tending to accumulate in localized regions. The bubble sizes varied widely, with some zones containing large coalesced bubbles and others being nearly bubble-free. This heterogeneity was especially prominent in the main channel and led to uneven mixing and the formation of stagnant zones, which can severely limit interfacial area and reduce both mass transfer efficiency and reaction uniformity.

Upon ultrasound activation, significant improvements in bubble morphology and distribution were observed. Large bubbles underwent fragmentation due to cavitation and acoustic streaming effects [39,40], resulting in the formation of uniformly dispersed microbubbles throughout the microchannel network (Fig. 7d). The presence of these fine bubbles increased the gas–liquid interfacial area, enhanced mass transfer rates, and facilitated more efficient mixing. Furthermore, their homogeneous spatial distribution reduced localized flow resistance, contributing to more stable, continuous, and predictable fluid transport.

To gain deeper insight into these effects, high-magnification observations were conducted at representative regions, including bifurcation junctions and the main trunk (Fig. 7b). Prior to ultrasound exposure, the fluid already contained a population of pre-existing bubbles, likely originating from dissolved gases or mechanical agitation during preparation (Fig. 7e). These bubbles were irregular in both size and distribution, potentially reducing the effective interfacial area available for mass transfer and limiting reaction efficiency. Following ultrasound treatment, these pre-existing bubbles were effectively broken down into smaller, more uniformly distributed microbubbles. This transformation not only increased the active interfacial area but may have also extended bubble residence time due to reduced buoyancy and improved suspension stability. Collectively, these changes led to enhanced mixing, improved reaction kinetics, and superior overall microreactor performance under ultrasonic excitation.

### 3.4 Tailoring the morphology and properties of CDs via a biomimetic leaf vein ultrasonic microreactor

To further validate the theoretical framework underpinning the structural optimization and acoustic energy transmission mechanisms of the ultrasound-assisted microreactor, we conducted a systematic experimental study using CD synthesis as a model reaction. Leveraging the biomimetic leaf-vein microchannel architecture, we achieved precise control over the morphology and optical properties of the synthesized CDs. This was confirmed through comprehensive characterization of the as-synthesized products under various ultrasonic conditions.

**Fig. 8:**
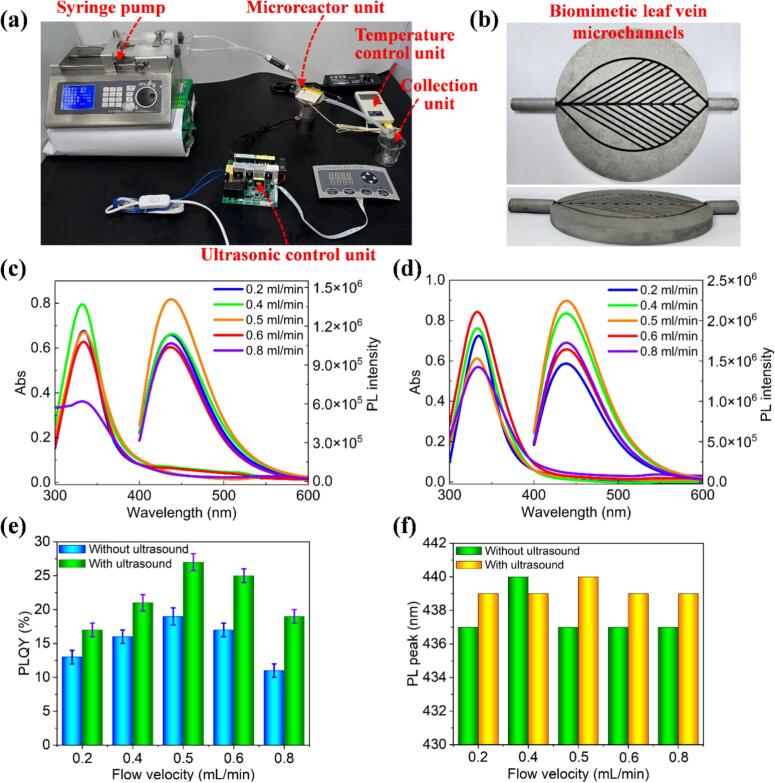
(a) Experimental setup of the ultrasonic microreactor platform. (b) Prototype of the biomimetic leaf-vein microchannel. UV–vis absorption and PL spectra of CDs synthesized at varying fluid velocities: (c) without ultrasonic treatment, (d) with ultrasonic treatment. (e) Comparison of the PLQY for CDs synthesized under different conditions. (f) Comparison of the PL peak positions for CDs synthesized under different conditions.

The ultrasonic microreactor experimental platform and biomimetic leaf-vein microchannel prototype are shown in Fig. 8, Fig. 8, respectively. Based on this system, the total reaction times are not more than 25 min (50 ml of solution). These reactions are much faster than most of the conventional methods (e.g., hydrothermal, solvothermal) [17].

A suite of analytical techniques—including UV–vis absorption spectroscopy, PL spectroscopy, TEM, and time-resolved fluorescence lifetime measurements—was employed to elucidate the formation dynamics and morphological evolution of CDs.

#### 3.4.1 Mechanism of biomimetic leaf vein ultrasonic microreactor in modulating the morphology and optical properties of indigo-emitting CDs

To investigate how the biomimetic leaf vein ultrasonic microreactor influences the morphology and optical properties of CDs and to validate the ultrasound enhancement effect, we focused on synthesizing indigo-emitting CDs. The flow velocity, a critical factor affecting CDs performance, was varied as the experimental variable. Other parameters were fixed as follows: the temperature was maintained at 190 °C, and the precursor concentration was set at 0.2 mol/L. The flow velocity within the microchannel was adjusted between 0.2 and 0.8 mL/min to evaluate its impact on CDs morphology, optical characteristics, and fluorescence lifetime under two conditions: without ultrasound (0 kHz) and with continuous ultrasound at 21 kHz.

As shown in Fig. 8, Fig. 8, the UV–vis absorption spectra of the synthesized CD solutions consistently display a primary absorption band within the 300–500 nm range. Regardless of flow velocity or ultrasound application, the first distinct absorption peak remains centered around 332 nm, corresponding to surface-state n–π* electronic transitions involving functional groups such as C=N and C=O [41]. This indicates that the core structure of the CDs remains chemically consistent across conditions. Under 365 nm UV excitation, the corresponding PL peak is centered around 437 nm for CDs synthesized without ultrasound and around 439 nm for those synthesized with ultrasound, confirming the successful synthesis of indigo-emitting CDs.

Specifically, as shown in Fig. 8, Fig. 8, both absorbance and PL intensity exhibit significant variations with changes in flow velocity, even when the CD concentration remains constant. At lower flow velocities (e.g., 0.2–0.6 mL/min), higher absorbance values (e.g., Abs = 0.62–0.79, without ultrasound) are observed, particularly in the UV region (300–400 nm), whereas at higher flow velocities (e.g., 0.8 mL/min, Abs = 0.36, without ultrasound), absorbance decreases. This trend is also reflected in the PL spectra, where PL intensity initially increases and then decreases with increasing flow velocity. Additionally, the PLQY of CDs synthesized under varying conditions is presented in Fig. 8e. The maximum PLQY reaches 19 % at a flow velocity of 0.5 mL/min without ultrasonic treatment, while ultrasonic treatment increases the maximum PLQY to 27 %. These findings suggest that slower flow velocities provide longer residence times, enhancing the interaction between the precursor and ultrasound. In contrast, higher flow velocities shorten the residence time and reduce the interaction with the acoustic field, thereby limiting reaction efficiency and resulting in lower PLQY. Another important finding is that, at the same flow velocity, ultrasound treatment significantly enhances the PLQY of the CDs, with an improvement of 42 %.

Under 365 nm UV excitation, when the flow rate varied between 0.2 and 0.8 mL/min, the CDs synthesized without ultrasonic treatment exhibited a peak shift of 3 nm. In contrast, the CDs synthesized with ultrasonic treatment showed a much smaller peak shift of only 1 nm. The average difference in peak position was 1.6 nm, suggesting that ultrasonic treatment does not significantly alter the luminescent core of the indigo-emitting CDs [42]. This behavior contrasts with previous reports on blue, green, and red CDs, where ultrasonic treatment has been shown to affect their optical properties [43]. Furthermore, the full width at half maximum (FWHM) of the PL spectra (Fig. S8) shows notable differences between the two conditions. In the absence of ultrasound, the FWHM values varied with flow velocity (e.g., 76–82 nm), implying a broader particle size distribution or the introduction of more surface defects as the flow velocity increases. This could be attributed to less uniform nucleation and growth under non-ultrasonic conditions. In contrast, under ultrasound, the FWHM values remain relatively stable (∼78 nm) across the range of flow velocities tested, indicating a more uniform particle size distribution and enhanced crystallinity. The stability in FWHM suggests that ultrasound helps to promote more consistent nucleation and growth, leading to nanoparticles with improved structural order and fewer defects. These findings highlight the dual role of ultrasound and flow velocity in modulating the formation of CDs. Ultrasound enhances the uniformity of energy distribution and promotes acoustic cavitation, which stabilizes the nucleation and growth processes of nanoparticles. Meanwhile, the flow velocity influences the residence time and mixing dynamics within the system, both of which are critical in determining particle size, structural order, and surface state distribution. Together, these factors emphasize the importance of precisely controlling both fluidic and acoustic parameters to achieve the desired nanoscale properties and consistent optical performance in carbon-based nanomaterials.

The photostability of CDs synthesized with and without ultrasonic treatment was evaluated by monitoring their fluorescence response under prolonged UV irradiation and subsequent thermal stress (Fig. S10). For CDs prepared at flow velocities of 0.2, 0.4, 0.5, 0.6, and 0.8 mL/min without ultrasonic treatment, the PL intensities retained ∼ 83.4 %, 84.3 %, 84.0 %, 84.4 %, and 81.8 % of their initial values, respectively, after 50 h of continuous exposure to 365 nm UV light at room temperature (Fig. S10a). Under identical conditions, CDs synthesized with ultrasonic treatment showed comparable performance, maintaining ∼ 84.8 %, 84.2 %, 84.6 %, 83.6 %, and 82.8 % of their initial intensities (Fig. S10b). These results confirm the excellent photostability of the CDs across different synthesis conditions and emphasize their promise for practical optoelectronic applications.

**Fig. 9:**
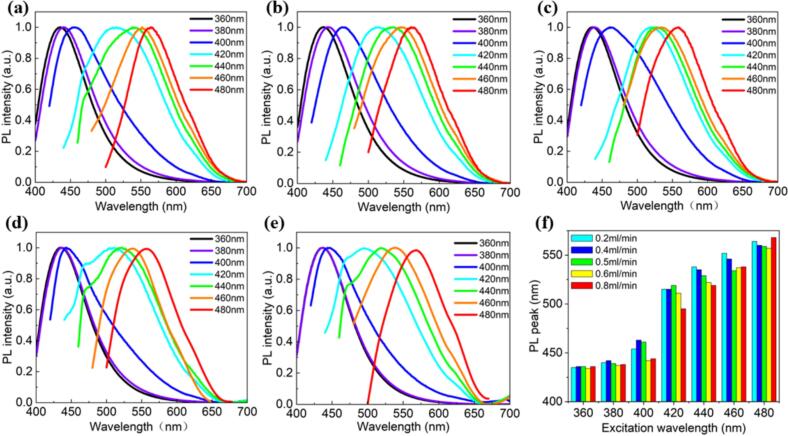
(a-e) PL evolution of CDs excited at wavelengths ranging from 360 to 480 nm, measured at flow velocities of (a) 0.2 mL/min, (b) 0.4 mL/min, (c) 0.5 mL/min, (d) 0.6 mL/min, and (e) 0.8 mL/min. (f) Plot showing the shift in PL peak positions as a function of the excitation wavelength.

We further explored the excitation-dependent and excitation-independent behaviors of the PL emission of the CDs synthesized without ultrasonic treatment, as illustrated in Fig. 9a-e. The results demonstrate that the PL spectra of the CDs, excited over a range of wavelengths from 360 to 480 nm, exhibit significant variations as a function of flow velocity (ranging from 0.2 to 0.8 mL/min). Specifically, at a flow rate of 0.2 mL/min, the emission peak positions under excitation wavelengths of 380, 400, 420, 440, 460, and 480 nm exhibited red shifts of 5, 7, 83, 104, 117, and 127 nm, respectively, compared to the 360 nm excitation (see Fig. 9a and Table S4). Furthermore, under excitation wavelengths ranging from 360 to 480 nm, the FWHM of the emission spectra exhibited an initial increase followed by a decrease, with values of 77.2 ± 1.5, 80.4 ± 1.6, 99.3 ± 2.5, 126.2 ± 3.0, 129.4 ± 3.0, 105.1 ± 2.6, and 85.7 ± 3.0 nm, respectively. When the flow rate was increased from 0.4 to 0.8 mL/min, the maximum red shifts observed were 128, 123, 123, and 134 nm, with the FWHM variation trend consistent with that observed at 0.2 mL/min (see Fig. 9b-e and Table S4). These findings underscore the strong dependence of the emission properties on both the excitation conditions and the flow dynamics within the system.

Fig. 9f specifically illustrates the relationship between the PL peak position and the excitation wavelength. As the excitation wavelength increases, a clear redshift of the PL peak is also observed, indicating a shift toward longer wavelengths. This redshift is a characteristic of excitation-dependent emission behavior, suggesting that the electronic structure or surface chemistry of the CDs is highly sensitive to both excitation conditions and varying flow velocities [44]. The observed shifts and intensity variations imply that changes in the fluid dynamics, such as flow velocity, can influence the nanoparticle morphology or the local environment surrounding the CDs, subsequently affecting their optical properties.

Additionally, we investigated the excitation-dependent behavior of the PL emission under ultrasonic conditions, as presented in Fig. S11a-f. As the excitation wavelength increases, a progressive redshift in the PL emission peak is observed. For example, at a flow rate of 0.2 mL/min, the emission peak positions under excitation wavelengths of 380, 400, 420, 440, 460, and 480 nm exhibited red shifts of 4, 23, 50, 99, 116, and 128 nm, respectively, compared to the 360 nm excitation (see Fig. S11a and Table S5). Additionally, under excitation wavelengths from 360 to 480 nm, the FWHM of the CDs' emission spectra followed an initial increase and subsequent decrease, with values of 78.0 ± 1.5, 82.3 ± 1.6, 92.4 ± 2.0, 113.1 ± 2.5, 122.9 ± 3.0, 96.8 ± 2.0, and 90.1 ± 2.0 nm, respectively. Notably, the FWHM values for CDs with ultrasonic treatment were smaller at the same excitation wavelengths compared to those without ultrasonic treatment, indicating that ultrasonic treatment influences the emission spectra of the CDs. Furthermore, when the flow rate was increased from 0.4 to 0.8 mL/min, the maximum red shifts observed were 115, 97, 83, and 80 nm, respectively. Similarly, compared to the samples without ultrasonic treatment, those with ultrasonic treatment exhibited smaller maximum red shifts (see Figs. S11b-e and Table S5). This excitation-dependent behavior is attributed to the interactions between the carbon bonds and surface functional groups within the CDs, without significant alterations in their chemical composition or nanoparticle size [45,46]. Therefore, the emission spectrum of the CDs can be finely tuned by varying the excitation wavelength or modulating the flow velocity, offering a versatile approach for controlling their optical properties.

**Fig. 10:**
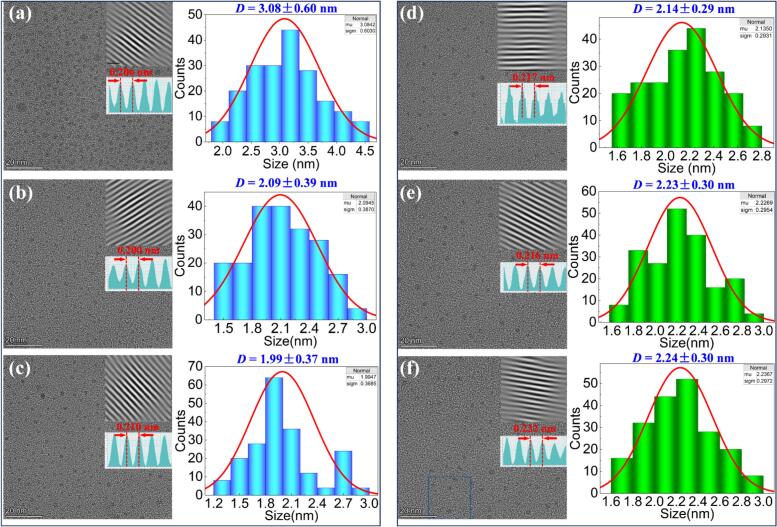
(a–c) TEM images and corresponding particle size distributions of CDs synthesized at flow velocities of (a) 0.2 mL/min, (b) 0.5 mL/min, and (c) 0.8 mL/min without ultrasonic treatment. Insets show HRTEM images and corresponding FFT patterns. (d–f) TEM images and particle size distributions of CDs synthesized at the same flow velocities, but with ultrasonic treatment.

The surface morphology and crystalline structure of the as-prepared CDs were characterized using TEM, as illustrated in Fig. 10. The results confirm that the CDs are well-dispersed and exhibit no signs of aggregation, predominantly maintaining a spherical shape. Fig. 10a-c show TEM images and particle size distributions of CDs synthesized at representative flow velocities of 0.2, 0.5, and 0.8 mL/min, respectively, without ultrasonic treatment. The corresponding average particle sizes are 3.08 ± 0.60 nm (Fig. 10a), 2.09 ± 0.39 nm (Fig. 10b), and 1.99 ± 0.37 nm (Fig. 10c). These results indicate that the CDs exhibit relatively broad size distributions, with a maximum standard deviation of 29 % in particle dimensions across all conditions. As the flow velocity increases, the particle size distribution becomes more heterogeneous, indicating a flow velocity-dependent variation in particle size.

High-resolution TEM (HR-TEM) images, shown in the insets, were analyzed using Fast Fourier Transform (FFT) and Inverse Fast Fourier Transform (IFFT). Multiple measurements of the interplanar spacing were taken, and the average value was calculated to ensure statistical reliability. The final interplanar spacings measured were 0.207 nm (Fig. 10a), 0.206 nm (Fig. 10b), 0.210 nm (Fig. 10c), 0.217 nm (Fig. 10d), 0.216 nm (Fig. 10e), and 0.232 nm (Fig. 10f) for CDs synthesized at flow velocities of 0.2, 0.5, and 0.8 mL/min, with and without ultrasonic treatment. Based on the characterization results, the materials exhibit a spherical morphology with well-defined lattice structures and no observable interlayer stacking, distinguishing characteristic of carbon-based materials. These features are more consistent with those of carbon quantum dots, rather than graphene quantum dots, which typically display a more layered or sheet-like structure [[47], [48], [49]]. Accordingly, the materials in this study are classified as carbon quantum dots, a distinct subclass of carbon dots, and will henceforth be referred to as 'CDs'.

Fig. 10d-f depict the morphology of cds synthesized at the same flow velocities (0.2, 0.5, and 0.8 mL/min) with ultrasonic treatment applied. The corresponding average particle sizes are 2.14 ± 0.29 nm (Fig. 10d), 2.23 ± 0.30 nm (Fig. 10e), and 2.24 ± 0.30 nm (Fig. 10f), with a maximum standard deviation of 2.7 % in particle dimensions across all conditions. TEM images reveal a more uniform particle size distribution, with a noticeable reduction in the overall particle size and a significantly narrower size range. These observations suggest that ultrasonic treatment significantly enhances particle size uniformity, resulting in a reduction in the average particle dimension. The enhanced uniformity and smaller size of the nanoparticles indicate that ultrasonic treatment plays a crucial role in promoting the formation of smaller and more consistent nanoparticles, which ultimately improves the overall quality, consistency, and crystallinity of the synthesized CDs.

To investigate the PL dynamics of the as-synthesized CDs, time-resolved PL decay measurements were conducted using a 375 nm pulsed laser as the excitation source. The PL decay curves of the six CD samples were analyzed and fitted to a double exponential decay model, as described by Equations (1), (2). The 95 % confidence intervals for *τ*_1_, *τ*_2_, and *τ*_avg._ were calculated from the covariance matrix of the nonlinear least-squares fitting. The goodness-of-fit was assessed using the fit residuals, reduced chi-square (χ^2^_red_), and Akaike Information Criteria (AIC), with the corresponding values provided in Table S6. These parameters confirm the reliability of the model selection. Additionally, all fluorescence decay curves were deconvoluted using the instrument response function (IRF), measured from Rayleigh scattering in a non-emissive reference sample (Fig. S12). Reconvolution fitting was then applied to accurately extract *τ*_1_, *τ*_2_, and *τ*_avg._.

**Fig. 11:**
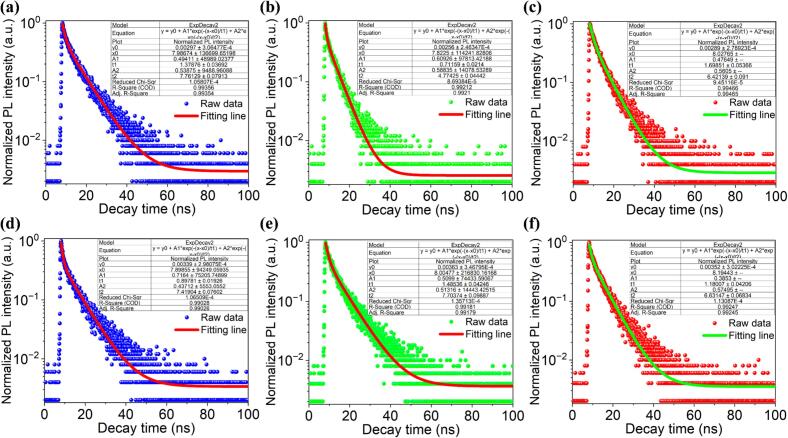
CDs time-resolved PL decay curve (raw data and fitting line) at different flow velocities without ultrasonic treatment: (a) 0.2 mL/min, (b) 0.5 mL/min, (c) 0.8 mL/min. CDs time-resolved PL decay curve at different flow velocities with ultrasonic treatment: (d) 0.2 mL/min, (e) 0.5 mL/min, (f) 0.8 mL/min.

As shown in Fig. 11a-f, the PL decay behavior of all six samples is similar, with two characteristic time constants, *τ*_1_ and *τ*_2_, observed across all CDs. These two components suggest that each sample contains multiple emitting species, each with distinct recombination rates [50]. The fast decay component, *τ*_1_, corresponds to radiative recombination from the intrinsic electronic states of the CDs, while the slower decay component, *τ*_2_, is associated with recombination processes occurring at the surface states [51]. This bifurcation in decay times highlights the complexity of the emission processes in CDs, driven by interactions within both the core and surface regions. The presence of both fast and slow decay components suggests that the emission properties of the CDs are significantly influenced by the local environment and surface states, which can be modulated through synthesis conditions, including ultrasonic treatment and flow velocity.

For CDs synthesized without ultrasonic treatment, the *τ*_1_ values measured at flow velocities of 0.2, 0.5, and 0.8 mL/min were 1.38 ns, 0.71 ns, and 1.70 ns, respectively, with corresponding *τ*_2_ values of 7.76 ns, 4.77 ns, and 6.42 ns. The average decay times (*τ*_avg._) for these samples were 7.61 ns, 4.44 ns, and 6.62 ns, respectively, as summarized in Table 5. In contrast, when ultrasonic treatment was applied, the decay dynamics exhibited notable changes. The *τ*_1_ values for CDs synthesized at the same flow velocities were 0.90 ns, 1.49 ns, and 1.18 ns, respectively, with *τ*_2_ values of 7.42 ns, 7.71 ns, and 6.63 ns. The corresponding *τ*_avg._ values for these samples were 6.78 ns, 7.59 ns, and 6.45 ns, respectively. The presence of both *τ*_1_ and *τ*_2_ in all samples suggests that the PL emission arises from two distinct luminescent centers. The first center is associated with π-π* transitions in the conjugated sp^2^ domains of the carbon core, while the second is attributed to n-π* transitions from oxygen- and nitrogen-containing surface functional groups [43]. These surface functional groups play a pivotal role in modulating the fluorescence properties of the CDs by influencing their emission characteristics.

**Table 5:** Fitted lifetimes of the CDs synthesized at different flow velocities without ultrasonic treatment or with ultrasonic treatment.

**External excitation**	**Flow velocities (mL/min)**	***A*_1_**	***τ*_1_ (ns)**	***A*_2_**	***τ*_2_ (ns)**	***τ*_avg._ (ns)**
Without ultrasonic treatment	0.2	0.49	1.38	0.54	7.76	7.61
	0.5	0.61	0.71	0.59	4.77	4.44
	0.8	0.48	1.70	0.56	6.42	6.62
With ultrasonic treatment	0.2	0.71	0.90	0.44	7.42	6.78
	0.5	0.51	1.49	0.51	7.71	7.59
	0.8	0.39	1.18	0.57	6.63	6.45

Additionally, the relatively stable *τ*_avg._ values across both ultrasonic and non-ultrasonic conditions suggest that these surface groups are the dominant factor governing the PL decay dynamics. The consistent behavior of the decay times across different synthesis conditions implies that the interactions between the surface functional groups and the carbon core are essential in determining the multicolor fluorescence observed in these materials. These interactions enable the tuning of the PL properties of the CDs, with contributions from both the core structure and the surface functionalities. Together, these factors interact synergistically to modulate the optical properties of the CDs, underscoring the complexity and versatility of their fluorescence behavior.

#### 3.4.2 The formation, development, and evolution of CDs' PL spectra in the ultrasonic microreactor

To investigate the formation, development, and evolution of CDs' PL spectra in the ultrasonic microreactor, we varied another key factor influencing CD performance—temperature—and conducted synthesis experiments for indigo-emitting CDs. UV–vis absorption spectra, as well as three-dimensional and two-dimensional PL spectra, were employed for characterization and analysis. The reaction temperatures tested ranged from 170 °C to 210 °C, all of which were effective for synthesizing high-quality CDs. In addition to temperature, other synthesis parameters, such as flow velocity, precursor concentration, and ultrasonic frequency, were optimized. The optimal values for these parameters were found to be 0.5 mL/min, 0.2 mol/L, and 21 kHz, respectively.

Fig. S13a shows the UV–vis spectra of the as-synthesized CDs without ultrasonic treatment. The first characteristic absorption band, typically observed between 300 and 400 nm, varies with reaction temperature. As the temperature increases, the absorption peak intensity initially rises to a maximum, then decreases at higher temperatures. Notably, a distinct valley appears in the absorption spectra at 200 °C and 210 °C, suggesting alterations in the electronic structure or surface functionalization of the CDs at these elevated temperatures. This behavior contrasts with the absorption properties of CDs synthesized at varying flow velocities, where no such valley feature was observed. Upon excitation with 365 nm ultraviolet light, the PL emission from the CDs synthesized at different reaction temperatures consistently exhibited a peak around 435 nm (Fig. S13b), indicating that the emission characteristics of the CDs, in the absence of ultrasonic treatment, remain relatively stable across the tested temperature range.

Upon applying ultrasonic treatment during the synthesis, significant changes in the absorption properties of the CDs were observed. As shown in Fig. S13c, the characteristic absorption band remained similar to that of the non-ultrasonic case for reaction temperatures below 200 °C. However, at temperatures above 200 °C, the absorption spectrum no longer exhibited the characteristic valley feature, suggesting a potential shift in the surface chemistry or structural arrangement of the CDs under ultrasonic influence. These changes imply that ultrasound may significantly affect the molecular structure or surface state of the CDs. Additionally, the PL emission of the CDs synthesized under ultrasonic conditions showed a shift in peak wavelength from 428 nm to 441 nm, depending on the reaction temperature (Fig. S13d). These shifts indicate that ultrasonic treatment alters the electronic structure of the CDs, thereby influencing their optical properties. The most notable result occurred at a reaction temperature of 190 °C, where the CDs exhibited the strongest absorption peak and the highest PL intensity. Furthermore, the PLQY measurements (see Fig. S14) revealed a maximum value of 20 % at a temperature of 190 °C without ultrasonic treatment, which increased to 27.5 % with ultrasound. Thus, this temperature appears to be the optimal condition for synthesizing CDs with enhanced optical properties, as compared to other temperatures where both absorption and PL intensities were significantly lower.

**Fig. 12:**
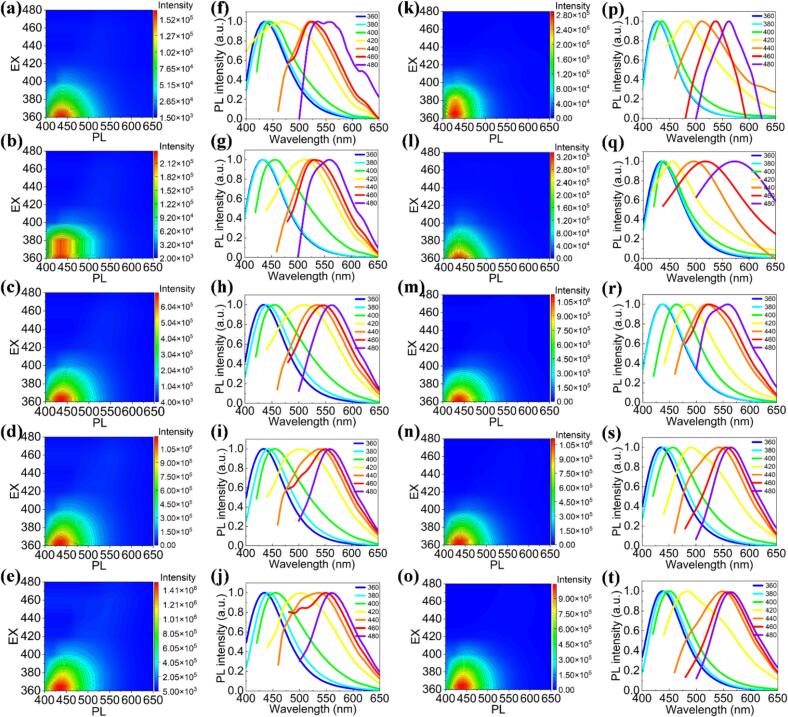
PL evolution of CDs synthesized at different temperatures, with and without ultrasonic treatment: 3D PL spectra of CDs synthesized without ultrasonic treatment at (a) 170 °C, (b) 180 °C, (c) 190 °C, (d) 200 °C, and (e) 210 °C. (f-j) Corresponding 2D normalized PL spectra under excitation wavelengths ranging from 360 to 480 nm. (k-o) 3D PL spectra of CDs synthesized with ultrasonic treatment at the same respective temperatures. (p-t) Corresponding 2D normalized PL spectra under identical excitation.

To further investigate the influence of reaction temperature and ultrasonic treatment on the optical properties of CDs, we systematically analyzed both the 3D PL spectra and 2D normalized PL spectra, as presented in Fig. 12. In the absence of ultrasonic treatment, the 3D PL spectra (Fig. 12a–e) demonstrate a progressive redshift and broadening of PL emission as the synthesis temperature increases from 170 °C to 210 °C. Specifically, at a temperature of 170 °C, the emission peaks of the CDs, without ultrasonic treatment, exhibited red shifts of 3, 7, 30, 86, 87, and 99 nm for excitation wavelengths of 380, 400, 420, 440, 460, and 480 nm, respectively, compared to the 360 nm excitation (see Fig. 12f and Table S7). Furthermore, under excitation wavelengths ranging from 360 to 480 nm, the FWHM of the CDs' emission spectra presented a trend of initial increase, followed by decrease, and then a subsequent increase, with values of 79.9 ± 1.5, 81.1 ± 2.2, 82.3 ± 2.4, 156.8 ± 3.5, 101.9 ± 2.4, 103.5 ± 2.6, and 133.8 ± 3.0 nm, respectively. When the temperature was increased from 190 °C to 210 °C, the maximum red shifts observed were 126, 127, 125, and 128 nm, respectively (see Fig. 12g-j and Table S7).

At 170 °C, the emission is characterized by a narrow width (see Fig. 12a), demonstrating strong excitation-wavelength dependence within a relatively short range of approximately 436–535 nm. As the temperature increases to 190 °C and beyond (Fig. 12c–e), the emission bands broaden and experience a redshift, extending to approximately 436–560 nm, accompanied by a reduced excitation dependence. This behavior suggests enhanced graphitization and greater heterogeneity of surface emissive states. The 2D normalized PL spectra (Fig. 12f–j) further support these observations, illustrating variations in emission intensity and peak positions with excitation wavelength. However, at 210 °C (Fig. 12j), a marked decline in PL intensity is noted, likely due to over-carbonization resulting in the formation of non-radiative surface defects that serve as trap states for excited carriers.

In contrast, ultrasonic-assisted synthesis significantly enhances the PL characteristics. Across all tested temperatures (Fig. 12k–o), the 3D PL contour plots exhibit sharper, more intense emission features with narrower FWHM (see Table S7), indicating improved uniformity in particle size and surface functionalization. Notably, CDs synthesized at 190 °C (Fig. 12, Fig. 12) display the most intense and symmetric emission centered around approximately 437 nm, suggesting that this temperature, in conjunction with ultrasonic treatment, achieves optimal structural and optical properties. The enhanced PL intensity and spectral resolution seen in the corresponding emission spectra (Fig. 12p–t) can be attributed to efficient precursor dispersion, controlled nucleation, and surface passivation facilitated by ultrasonication, which collectively mitigate defect-induced non-radiative recombination.

The pronounced redshift observed in the PL peaks corresponding to the 420 nm excitation wavelength, as shown in Fig. 12f-j and 12p-t, can be attributed to several factors. One significant influence is the dynamics of the excited state, including carrier trapping and recombination processes, which facilitate energy relaxation and result in emission at longer wavelengths. Additionally, increased excitation intensities may induce thermal effects, leading to localized heating that alters the material's electronic structure.

Overall, these observations conclusively demonstrate that ultrasonic treatment significantly enhances both the PL intensity and spectral uniformity of CDs across all tested temperatures, with the most substantial improvements evident at 190 °C. These findings highlight the critical role of sonochemical effects in modulating the electronic structure and emissive properties of CDs, thereby presenting an effective approach for optimizing their optoelectronic performance through advanced process engineering.

## 4 Conclusions

In this work, we report the development of a high-performance ultrasonic microreactor inspired by biomimetic leaf vein architectures, enabling the rapid and efficient synthesis of indigo-emitting CDs. To improve flow field uniformity and elucidate transport dynamics within the microchannel, we established a COMSOL Multiphysics–based optimization framework. Guided by numerical simulations and validated experimentally, the optimal microchannel configuration was identified: a Type III leaf vein profile, a fractal angle of 30°, a depth-to-width ratio of 3, and a single-inlet design. These parameters collectively enhance flow uniformity while minimizing energy dissipation.

To further maximize acoustic energy transfer, the ultrasonic transducer was directly integrated with the microreactor. Systematic simulations revealed that an operating frequency of 21 kHz and a power input of 100 W deliver the most efficient performance. Notably, the experimentally determined resonance frequency of 21.5 kHz closely matched the simulated value (21 kHz), confirming the predictive accuracy of our modeling framework. Visualization experiments corroborated these findings, demonstrating that ultrasonic irradiation fragmented large bubbles into uniformly dispersed microbubbles, thereby increasing interfacial area, extending bubble residence time, and stabilizing suspension behavior.

Using this optimized system, we achieved the synthesis of indigo-emitting CDs with a maximum PLQY of 27.5 % and a narrow FWHM of ∼ 78 nm under 365 nm excitation. The reaction time was less than 25 min for a 50 mL solution, representing a substantial improvement in efficiency compared with conventional methods. Complementary characterization techniques—including UV–vis absorption, PL spectroscopy, TEM, and fluorescence lifetime analysis—revealed that the interplay of flow velocity, reaction temperature, and ultrasonic modulation governs CD nucleation and growth, thereby dictating their structural and optical properties. These results establish ultrasound as a powerful tool for regulating CD synthesis and tailoring their photophysical behaviour.

Overall, this study highlights the synergistic coupling of ultrasonic energy, biomimetic microreactor design, and simulation-guided optimization in achieving uniform, high-quality CDs. The demonstrated strategy provides a versatile platform for scalable, high-throughput production of carbon-based nanomaterials with potential applications in optoelectronics, biomedicine, sensing, and artificial photosynthesis.

Looking forward, future efforts should address the challenges of scaling up to industrial production, particularly with respect to long-term reactor stability under continuous flow conditions. Further optimization of precursor chemistry, reaction parameters, and cavitation dynamics will be essential to enhance CD yield, PLQY and functional versatility. Moreover, a deeper mechanistic understanding of multiphase interactions in ultrasonic microreactors could enable precise control over reaction kinetics and material properties, advancing both fundamental science and large-scale applications.

## CRediT authorship contribution statement

**Longshi Rao:** Writing – review & editing, Writing – original draft, Methodology, Funding acquisition, Conceptualization. **Shengxin Zhu:** Visualization, Methodology, Investigation, Data curation. **Jiaying Liu:** Visualization, Conceptualization. **Qiuling Bai:** Visualization, Data curation. **Junxian Zou:** Visualization, Investigation, Data curation. **Chuheng Deng:** Visualization, Data curation. **Hongze Tu:** Investigation. **Qingxian Liu:** Writing – review & editing, Visualization, Funding acquisition, Data curation. **Guisheng Zhong:** Writing – review & editing, Investigation, Funding acquisition, Data curation. **Xiaodong Niu:** Validation, Supervision, Methodology. **Jiasheng Li:** Supervision, Conceptualization.

## Declaration of competing interest

The authors declare that they have no known competing financial interests or personal relationships that could have appeared to influence the work reported in this paper.
