# Supplementary material for: Bio-inspired ultrasonic microreactor for efficient synthesis of indigo-emitting carbon dots with tunable morphology and enhanced optical properties
Source: Ultrason Sonochem. 2025 Sep 17;121:107576. doi: 10.1016/j.ultsonch.2025.107576 (PMC12482640; doi:10.1016/j.ultsonch.2025.107576)
Supplement: Supplementary Data 1 [file mmc1.docx]

**Supplementary Information**

**Bio-inspired ultrasonic microreactor for efficient synthesis of indigo-emitting carbon dots with tunable morphology and enhanced optical properties**

**Longshi Rao^1,2,3*^, Shengxin Zhu^1,2^, Jiaying Liu^1,2^, Qiuling Bai^1,2^, Junxian Zou****^1,2^, Chuheng Deng^1,2^, Hongze Tu^1,2^, Qingxian Liu^1,2^, Guisheng Zhong^1,2,4*^, Xiaodong Niu^1,2^ and Jiasheng Li^5^**

^1^ Department of Mechanical Engineering, College of Engineering, Shantou University, Shantou 515063, China

^2^ Intelligent Manufacturing Key Laboratory of Ministry of Education, Shantou University, Shantou 515063, China

^3^ Guangdong Provincial Key Laboratory of Automotive Display and Touch Technologies, Shantou Goworld Display Technology Co., Ltd., Shantou 515041, China

^4^ Shantou Key Laboratory for Intelligent Equipment and Technology, Shantou University, Shantou 515063, China

^5^ National & Local Joint Engineering Research Center of Semiconductor Display and Optical Communication Devices, South China University of Technology, Guangzhou, 510641, China

*Corresponding author: lsrao@stu.edu.cn; gszhong@stu.edu.cn

**SI 1. Non-dimensional numbers**

**(1) Mean velocity (*U*)** is determined by:

*U* = *Q*/(*D·h*)

Where *Q* is the volumetric flow rate, *D* and *h* is the width depth of the channel inlet, respectively.

**(2) Reynolds number (*R*_e_)** is calculated using the formula:

*R_e_* = *ρ*⋅*U*⋅*D_h_*/*μ*

where *ρ* is the density of DMF, *D_h_* is the hydraulic diameter, and *μ* is the viscosity of DMF.

**(3) Peclet number (*P*_e_)** is given by:

*P_e_* = *U·L/D_I_*

where *L* is the characteristic channel length related to the diffusion of solutes, *D_I_* is the representative molecular diffusivity for precursor/solute (sensitivity to 10⁻⁸–10⁻¹⁰).

**(4) Weber number (*W*_e_)** is *d*efined as:

*W_e_ = ρ·U^2^·D_h_​/σ*

where *σ* is the surface tension of the fluid.

**(5) Cavitation number (*C*_a_)** can be determined using:

*C_a_ = μ·U/σ*

**(6) Strouhal number (*S*_t_)**, applicable in our mixing analysis, is defined by:

*S_t_* = *f·D_h_*​/*U*

where *f* is a frequency characteristic of the cavitation and *L* is a characteristic length.

Based on the properties of the solvent and the geometry described in the manuscript, we have computed key dimensionless numbers across the experimental flow range of 0.2–0.8 mL min⁻¹. We have explicitly stated our assumptions and provided full step-by-step calculations, along with a summary for each flow rate at the two channel depth extremes (*h* = 0.8 mm and 2.4 mm), which are included in the Supplementary Information. Below, we summarize the assumptions, the calculated ranges, and a brief mechanistic interpretation. Since the properties of DMF dominate the mixture, the non-dimensional numbers were calculated using DMF as follows:

**(1) Assumptions / input**

- Solvent approximated by DMF-rich properties (as requested): *ρ* = 1.087×10³ kg·m⁻³; *μ* = 0.944×10⁻³ Pa·s (25 °C).
- Channel inlet: width *D* = 1.44 mm (0.00144 m); depth *h* = 0.8 – 2.4 mm (0.0008 –0.0024 m).
- Volumetric flow rates *Q* = 0.2, 0.5, 0.8 mL·min⁻¹.
- Channel length used for *P*_e_: *L* = 0.10 m (stated for reproducibility).
- Representative molecular diffusivity for precursor/solute: *D_I_* = 1.0×10⁻⁹ m²·s⁻¹ (sensitivity to 10⁻⁸–10⁻¹⁰).
- Surface tension (DMF/water mixture, assumed): *σ* = 0.04 N·m⁻¹.
- Ultrasound frequency for Strouhal estimate: *F_f_* = 21 kHz (typical lab transducer).
- Hydraulic diameter (rectangular): *D_h_*_​_ = 2*D·h*/(*D* + *h*).
- Formulas used: *U* = *Q*/(*D·h*); *R_e_* = *ρ·U·D_h_*​/*μ*; *P_e_* = *U·L*/*D_I_*; *W_e_* = *ρ·U*^2^*·D_h_*​/*σ*; *C_a_* = *μ·U*/*σ*; *S_t_* = *f·D_h_*​/*U*; Streaming Reynolds *R_es_*​ ≈ *U_s_*​*·D_h_*_​_/*ν* (requires an acoustic streaming velocity amplitude *Us*​).

**(2) Representative computed values**

- Hydraulic diameter *D_h_*​:
- h = 0.8 mm → *D_h_*​ = 1.03×10⁻³ m;
- h = 2.4 mm → *D_h_*​ = 1.80×10⁻³ m;
- Mean velocity *U* (m·s⁻¹):
- h = 0.8 mm: 0.00289 → 0.01157 (*Q* = 0.2 → 0.8 mL·min⁻¹);
- h = 2.4 mm: 0.00096 → 0.00386;
- Dimensionless ranges (*Q* = 0.2 → 0.8 mL·min⁻¹):
- Reynolds number, *R*_e_= *ρ·U·D_h_*​/*μ*: ≈ 1.99 → 13.7 (all cases *R*_e_ ≪ 2,300).
  *Conclusion:* bulk flow is laminar for every tested condition.
- Péclet number, *P*_e_ = *U·L/D_I_*: ≈ 9.6×10⁴ → 1.16×10⁶ (for *D_I_* = 1×10⁻⁹ m²·s⁻¹).
  *Conclusion:* *P*_e_ ≫ 1 → axial transport is overwhelmingly convective; molecular diffusion is negligible on the reactor length scale.
- Weber number, *W_e_* = *ρ·U*^2^*·D_h_*​/*σ*: ≈ 2.3×10⁻⁴ → 3.7×10⁻³ (all cases *W_e_* ≪ 1).
  *Conclusion:* steady mean flow inertial stresses are small relative to surface tension — mean flow alone would not induce inertial bubble breakup.
- Capillary number, *C_a_* = *μ·U*/*σ*: ≈ 6.8×10⁻⁵ → 2.7×10⁻⁴ (order 10⁻⁵–10⁻⁴).
  *Conclusion:* viscous deformation due to mean flow is negligible compared with interfacial tension.
- Strouhal number, *S_t_* = *f·D_h_*​/*U*: ≈ 1.8×10³ → 7.1×10³ (*h* = 0.8 mm) and 9.3×10³ → 3.7×10⁴ (*h* = 2.4 mm).
  *Conclusion:* St ≫ 1 — ultrasonic oscillations act on a timescale much faster than convective passage across the hydraulic diameter (rapid unsteady forcing of interfaces).
- Streaming / acoustic Reynolds (order estimate): *R_es_*​ ≈ *U_s_*​*·D_h_*​/*ν*. Using plausible streaming velocities *U_s_*_​_ = 0.001–0.05 m·s⁻¹ (typical lab streaming amplitudes reported in the literature) and *ν* = *μ*/*ρ* ≈ 8.69×10⁻⁷ m²·s⁻¹ yields *R_es_*​ ≈ 1–103 (h = 0.8–2.4 mm, depending on *U_s_*).
  *Conclusion:* realistic acoustic streaming amplitudes produce local *R_es_*_​_ large enough to generate vortices and strong micro-mixing even though bulk *R*_e_ is small.


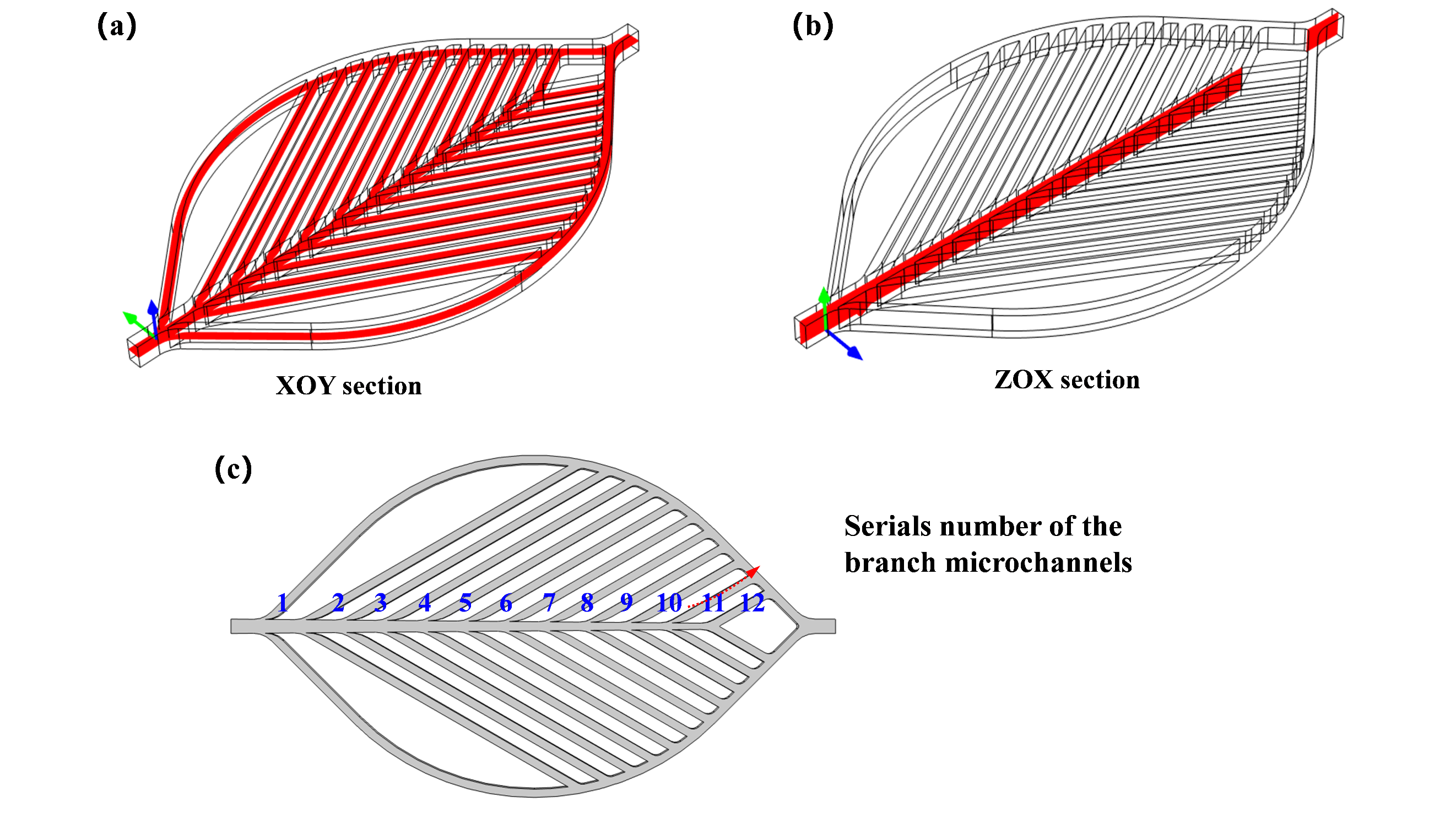


**Fig. S1.** (a) XOY plane: Top view of the microchannel. (b) ZOX plane: Front view of the main channel. (c) Labeling scheme for the branching channels.


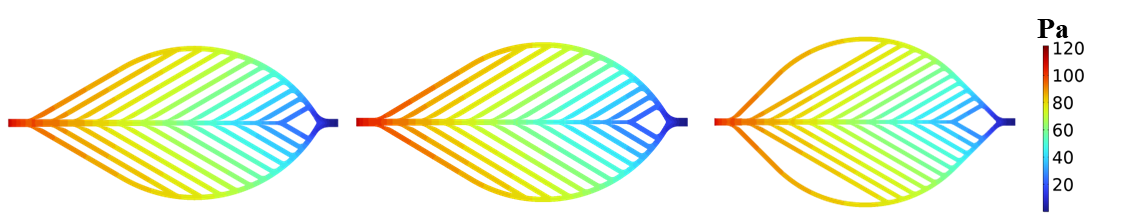


**Fig. S2.** Pressure distribution contour maps of the internal branching channels within the microchannel for different vein profile structures in the XOY plane.

**Table S1.** Streamline distribution contour maps of the internal branching channels within the Type III microchannel at different depth-to-width ratios in the XOY plane.


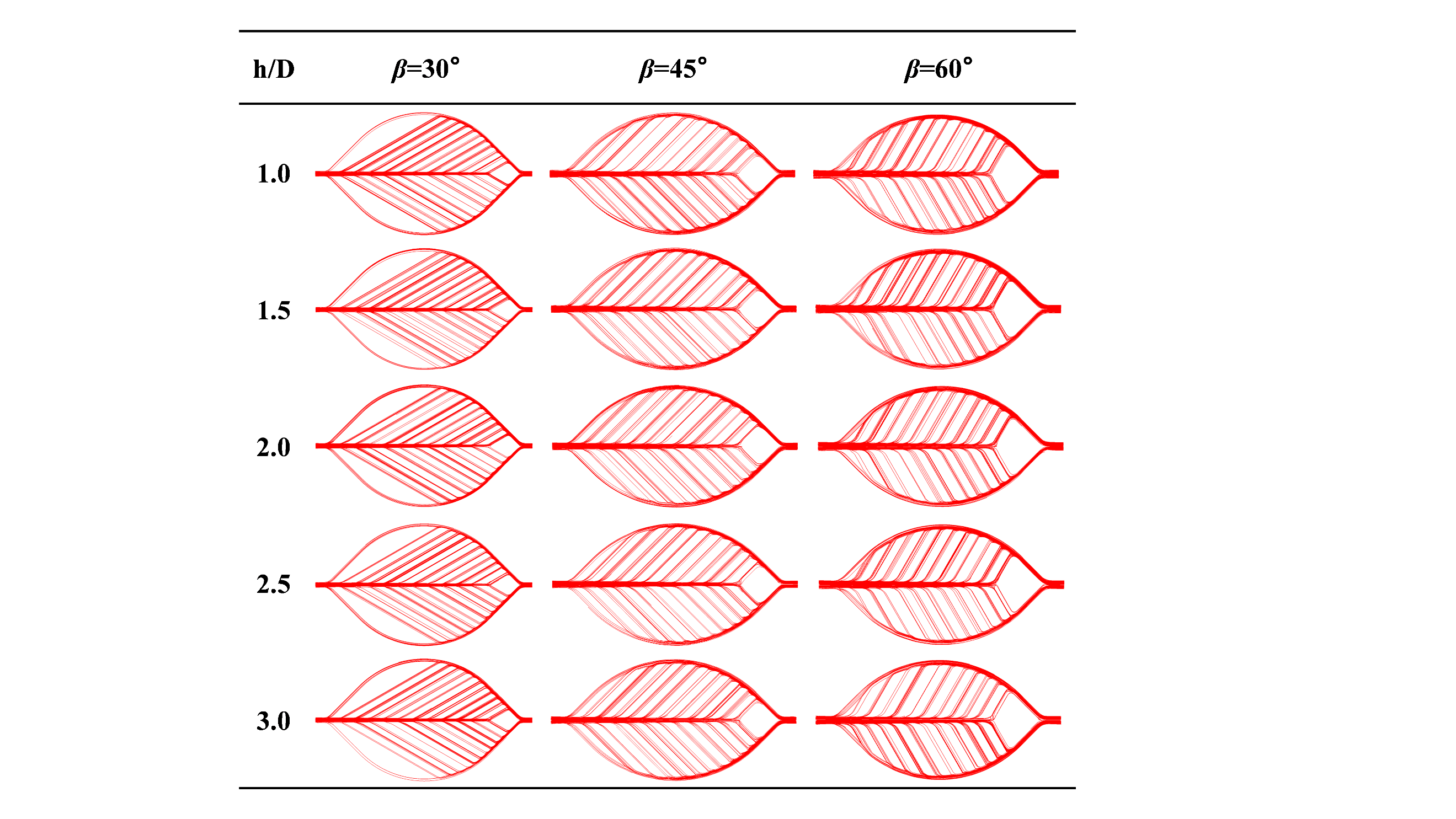


**Table S2.** Pressure distribution contour maps of the main and branching channels within the Type III microreactor at different depth-to-width ratios in the XOY plane.


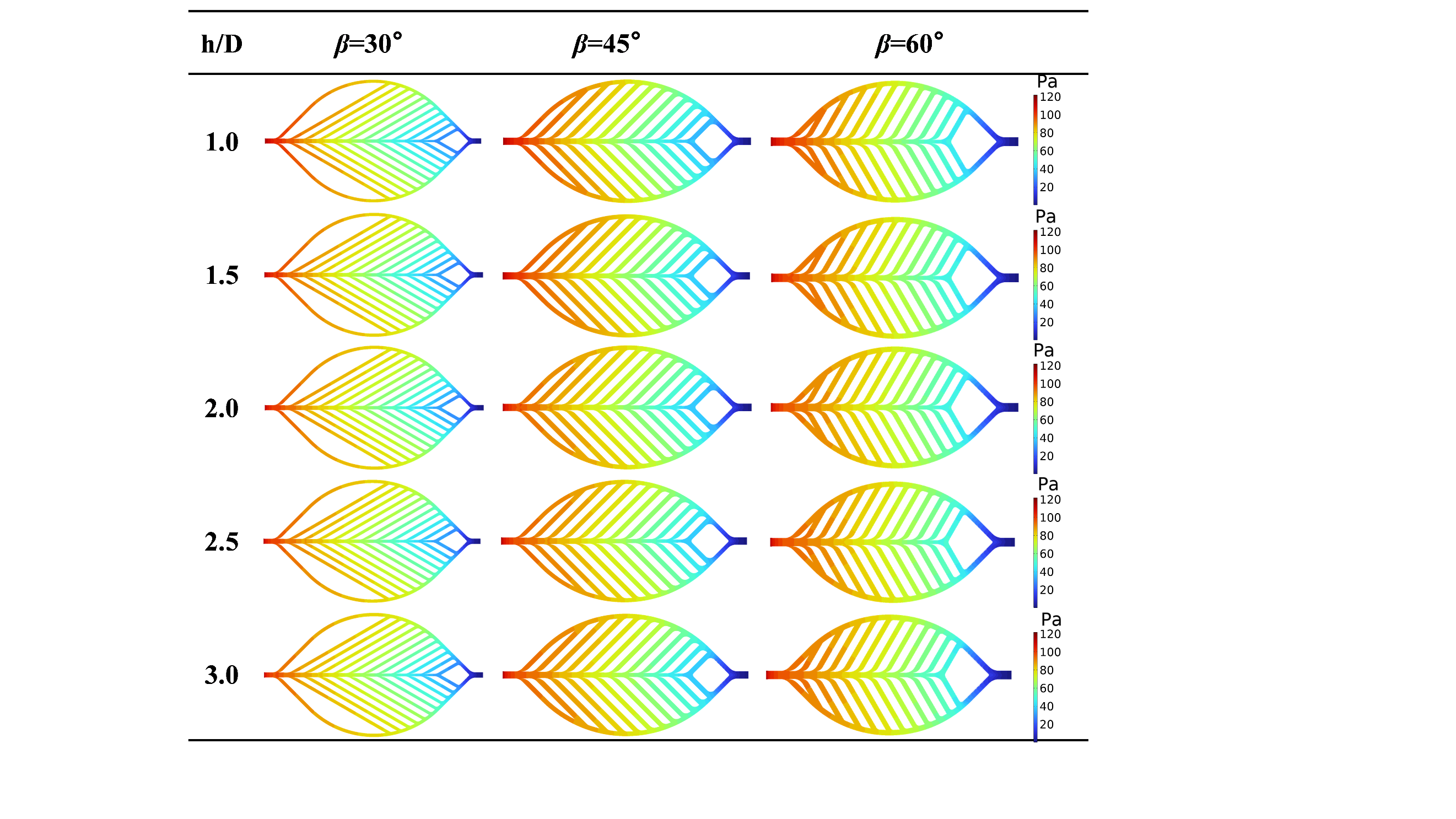


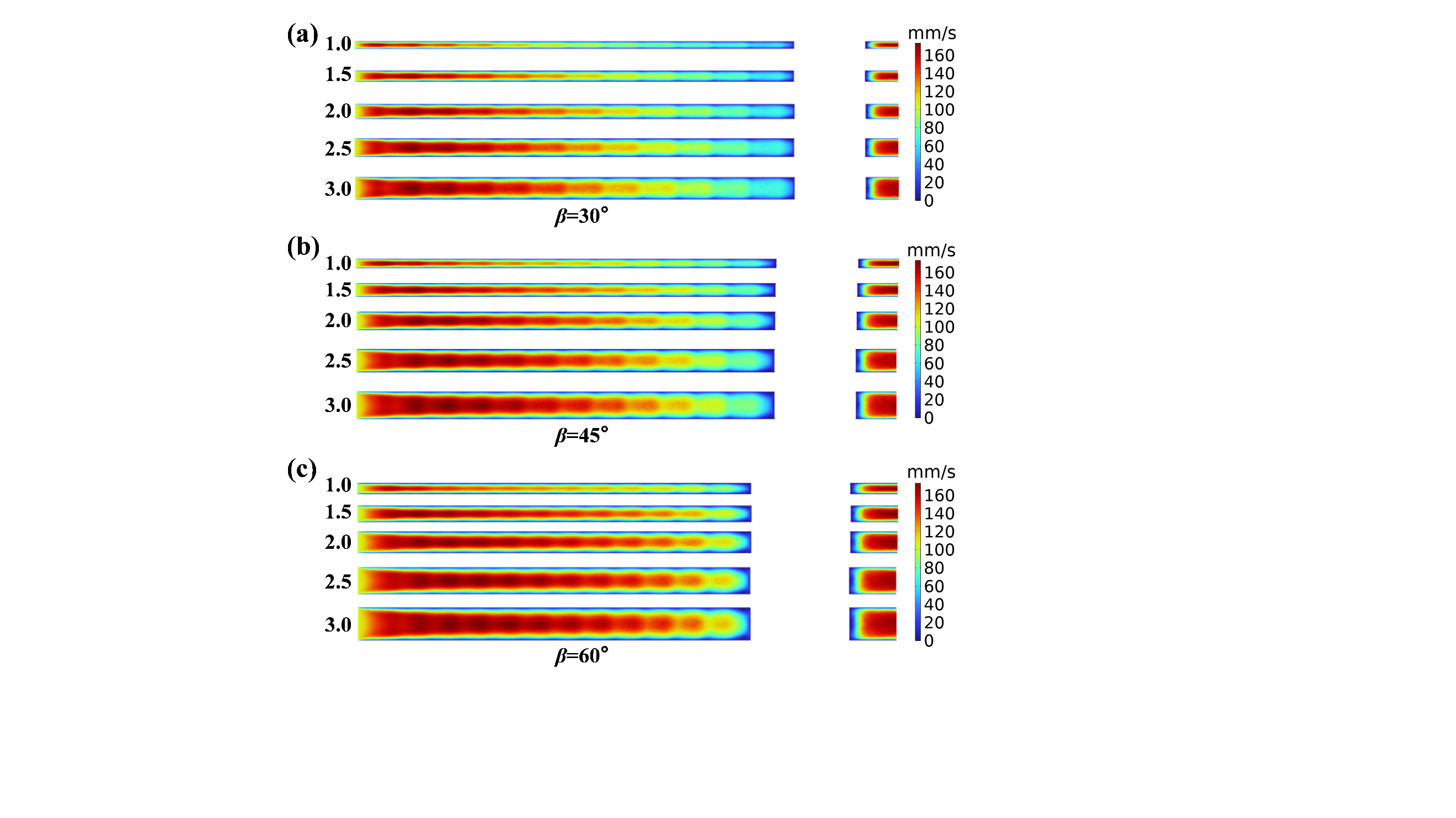


**Fig. S3.** Velocity distribution of the main channel in the microreactor at different depth-to-width ratios and fractal angles in the ZOX plane: (a) 30° fractal angle, (b) 45° fractal angle, (c) 60° fractal angle.

**Table S3.** Streamline distribution contour maps of the internal branching channels within the double-inlet or single-inlet Type III microreactor at different depth-to-width ratios in the XOY plane.


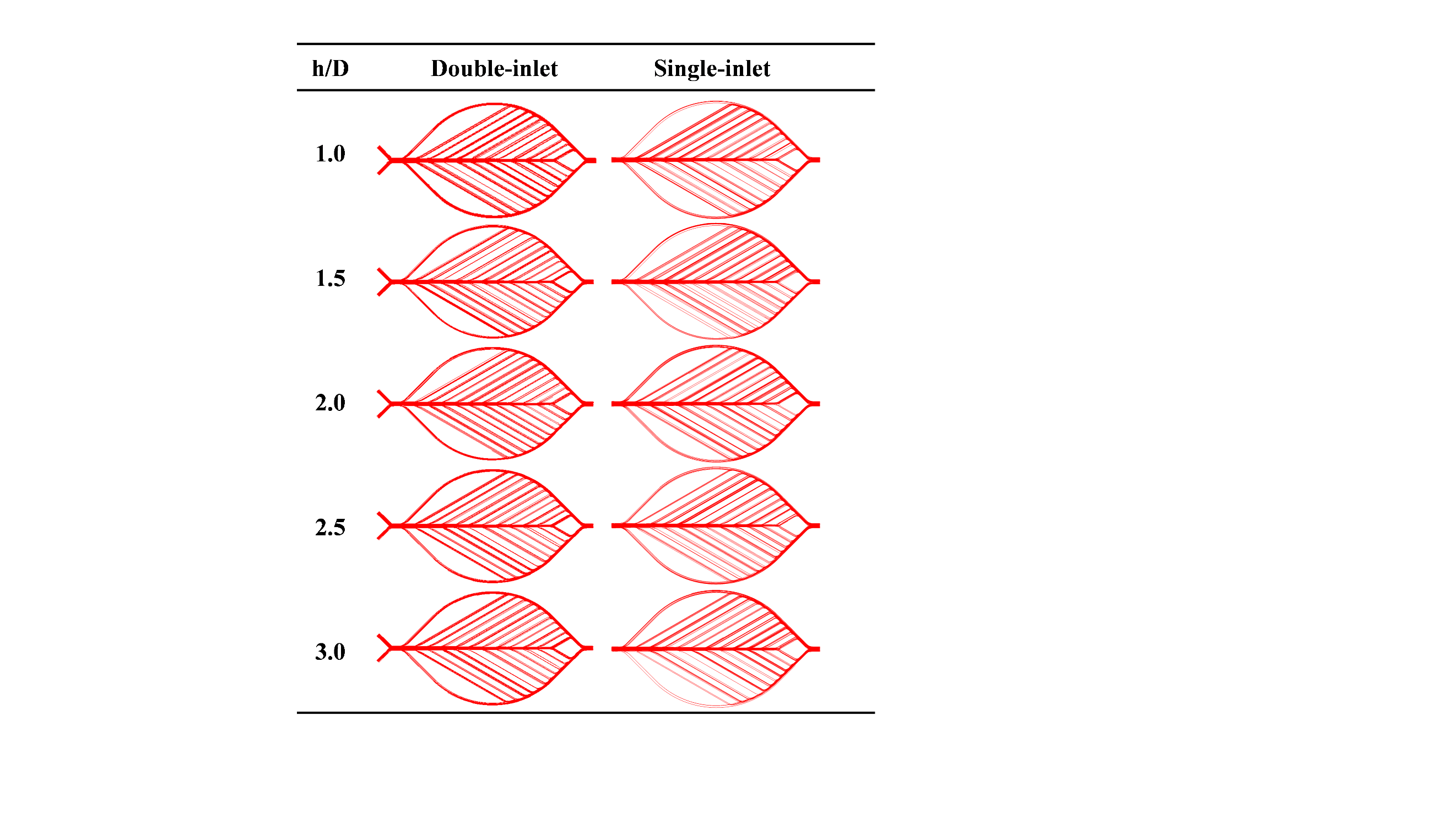


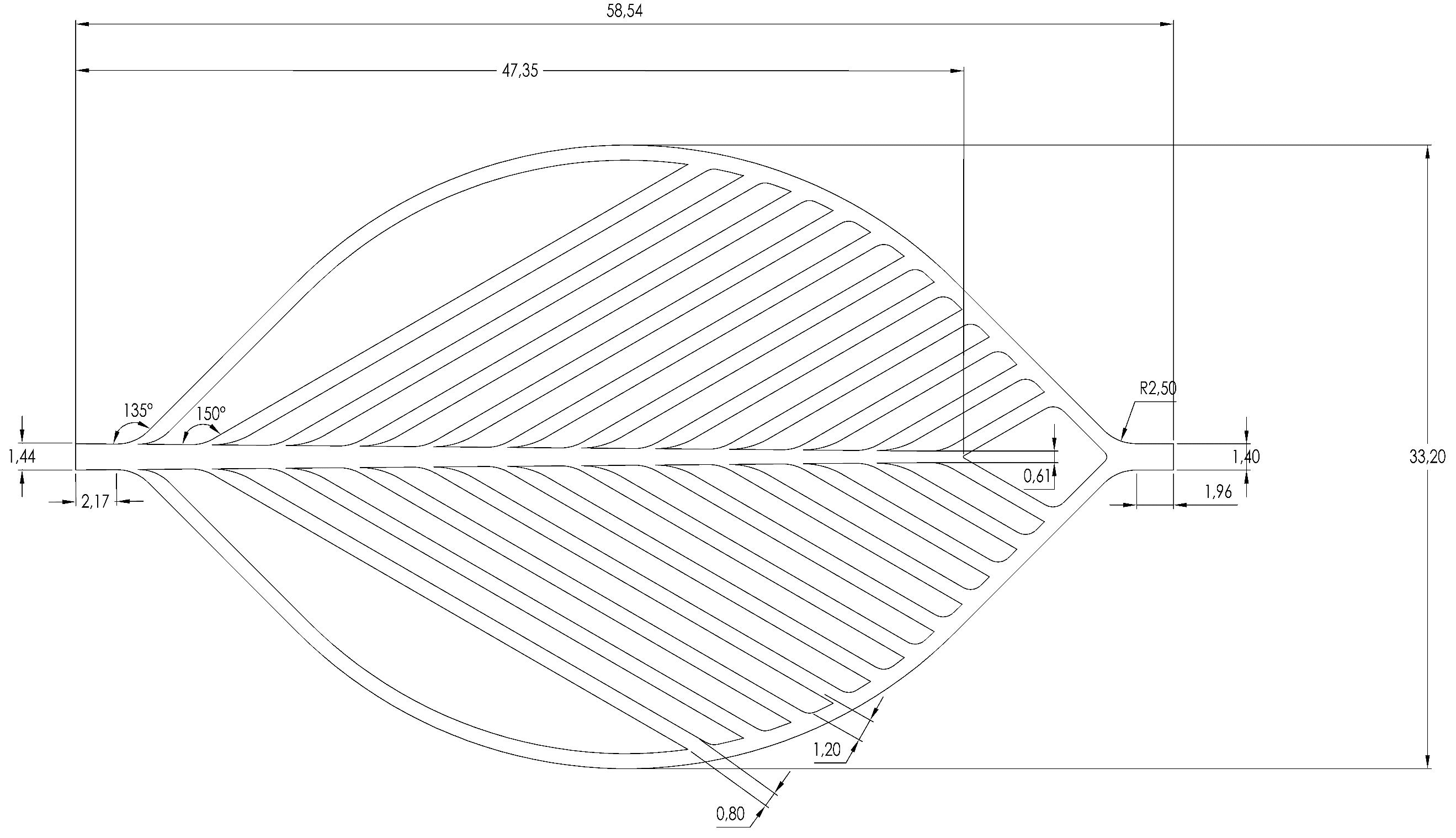


**Fig. S4**. Optimized key dimension annotation diagram of the Type III microchannel (unit: mm). The depth ranges from 0.8 to 2.4 mm.


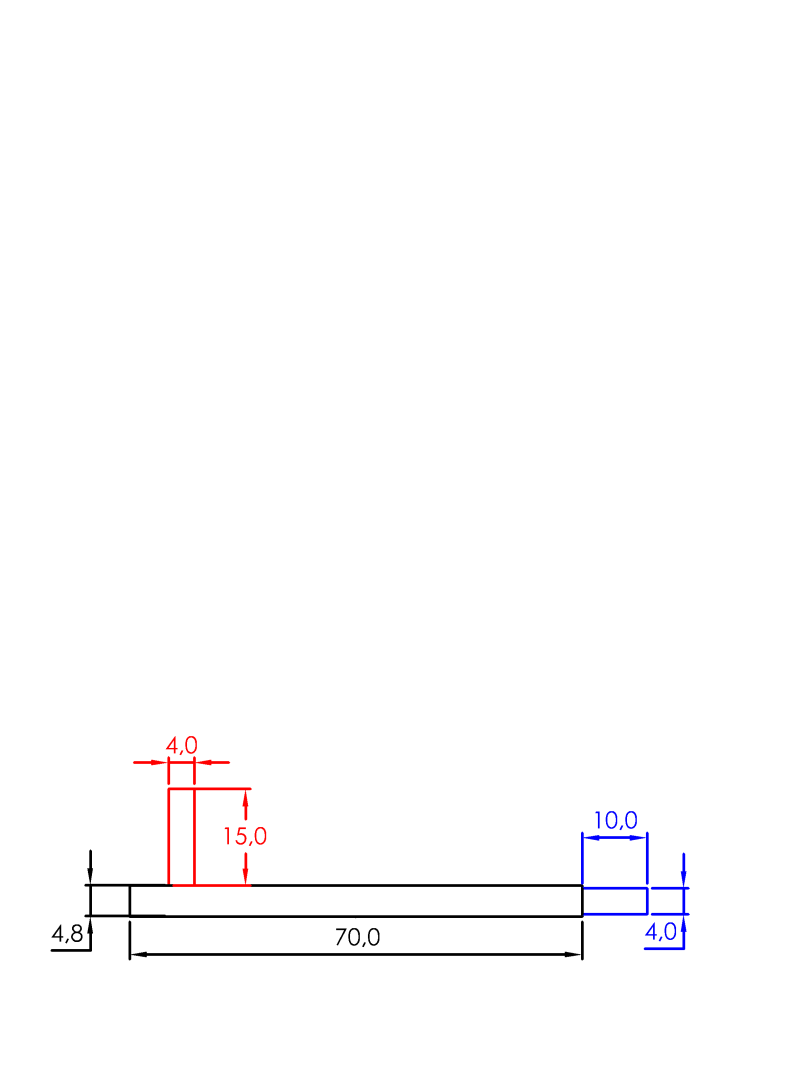


**Fig. S5.** Design diagram of the biomimetic leaf vein microreactor.


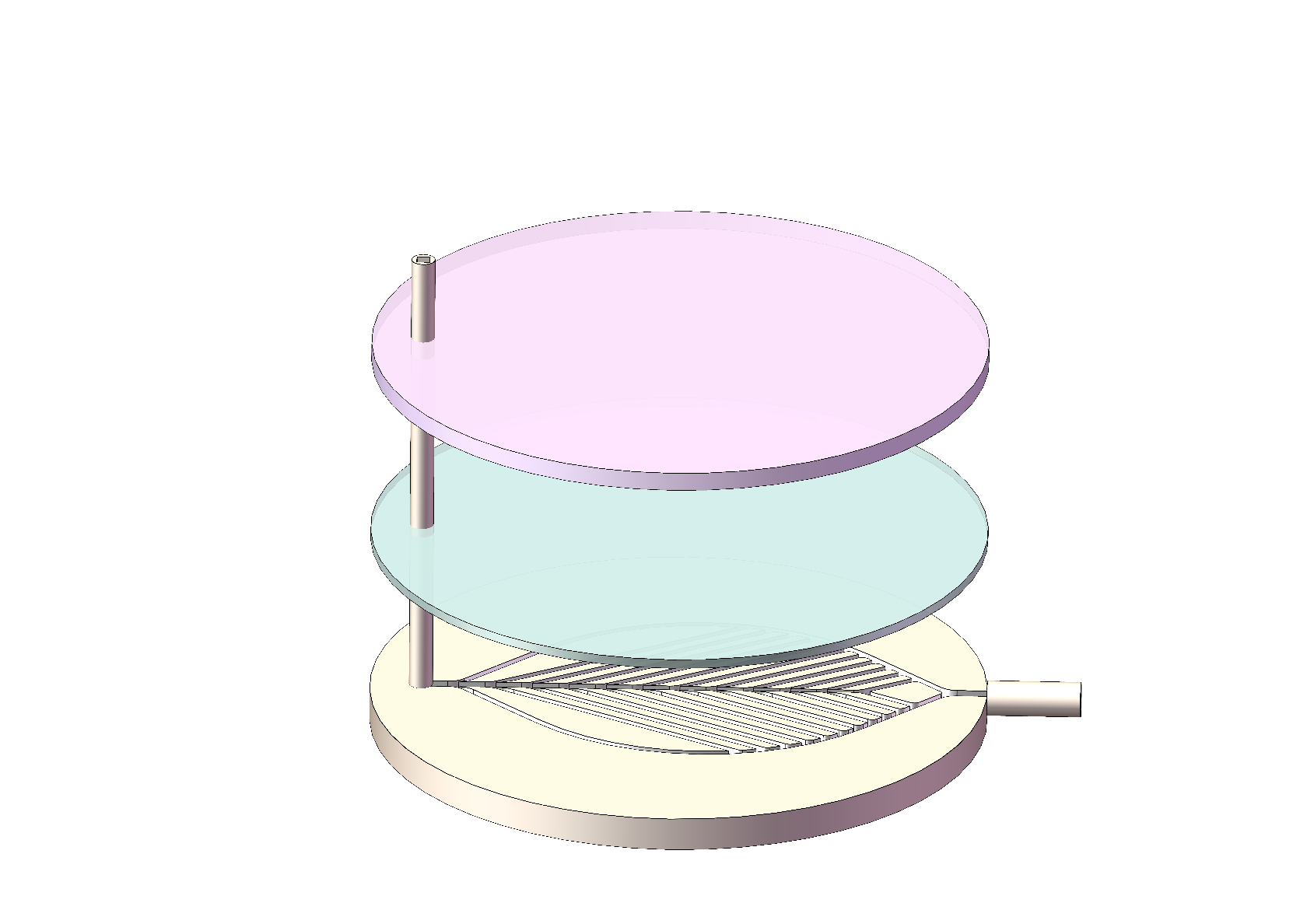


**Fig. S6.** Exploded view of the structure of the biomimetic leaf vein microreactor.


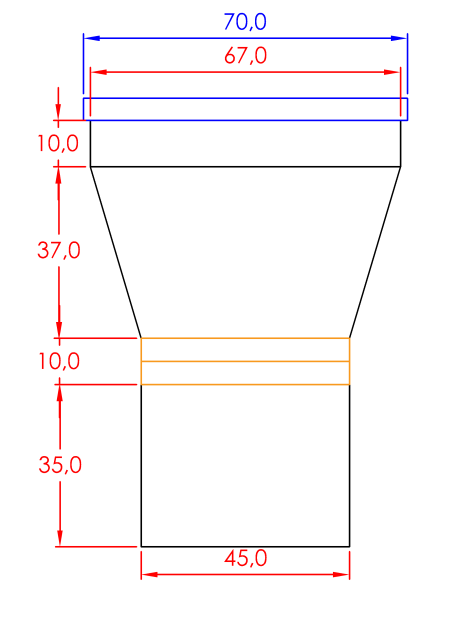


**Fig. S7.** Design diagram of the ultrasonic microreactor with a resonant frequency of 21 kHz and a rated power of 100 W.


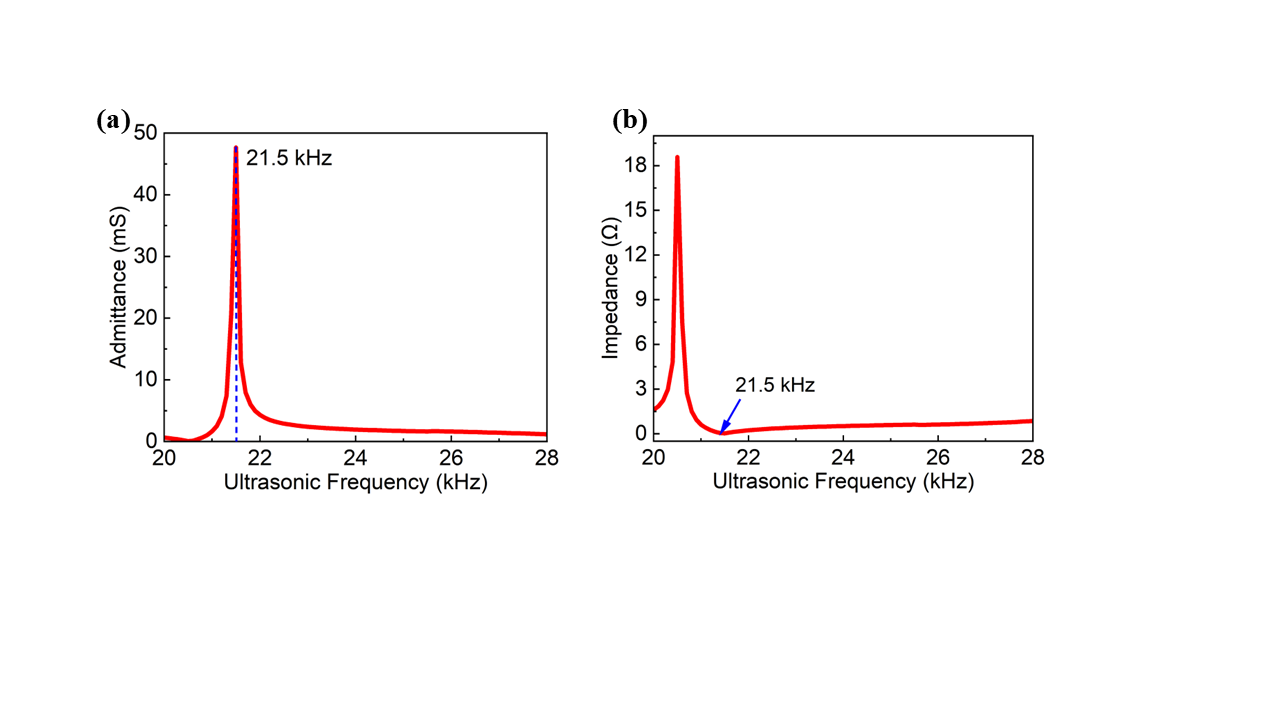


**Fig. S8.** (a) Admittance curve and (b) impedance curve of the ultrasonic microreactor.


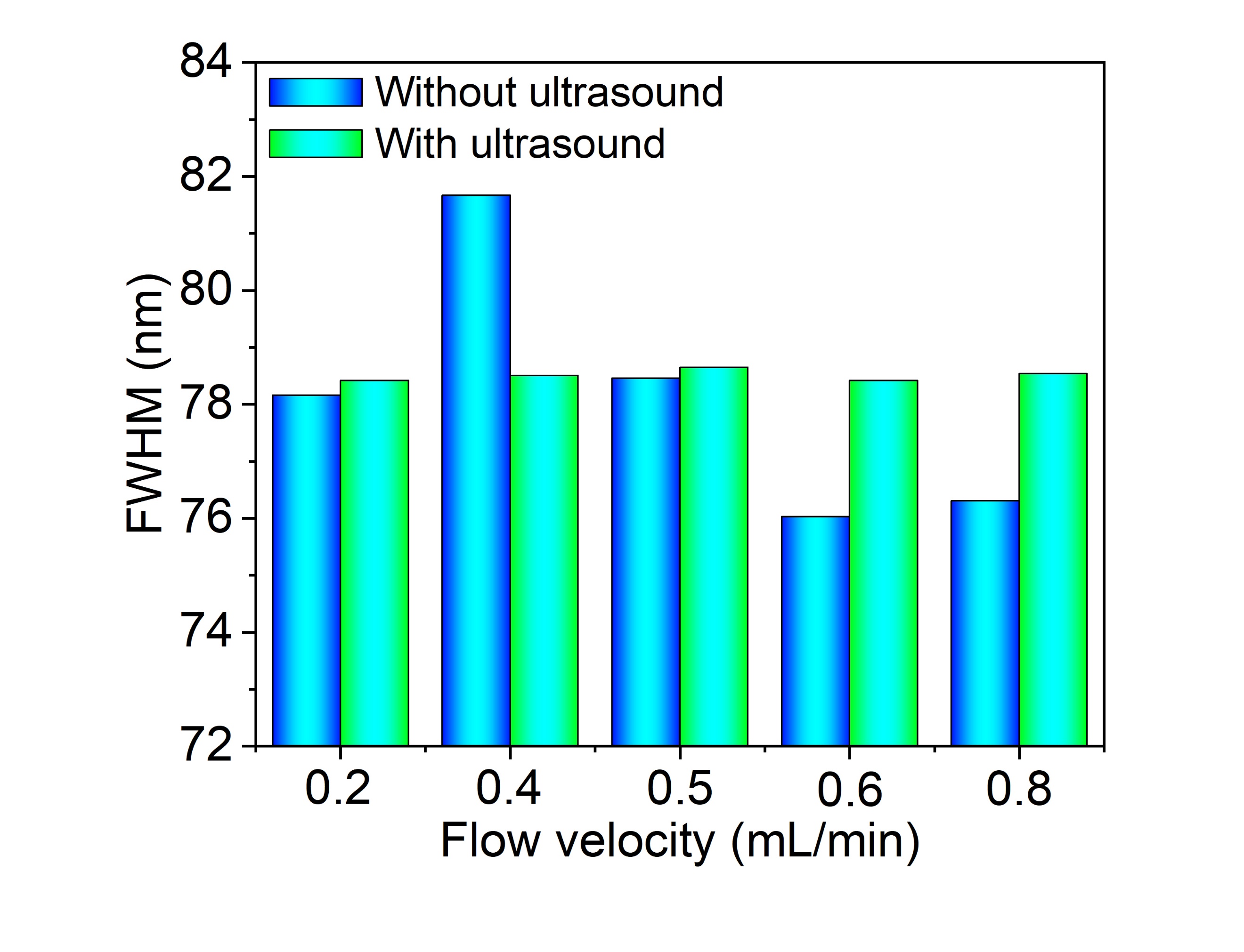


**Fig. S9.** Comparison of the FWHM values of the PL spectra before and after ultrasonic treatment.


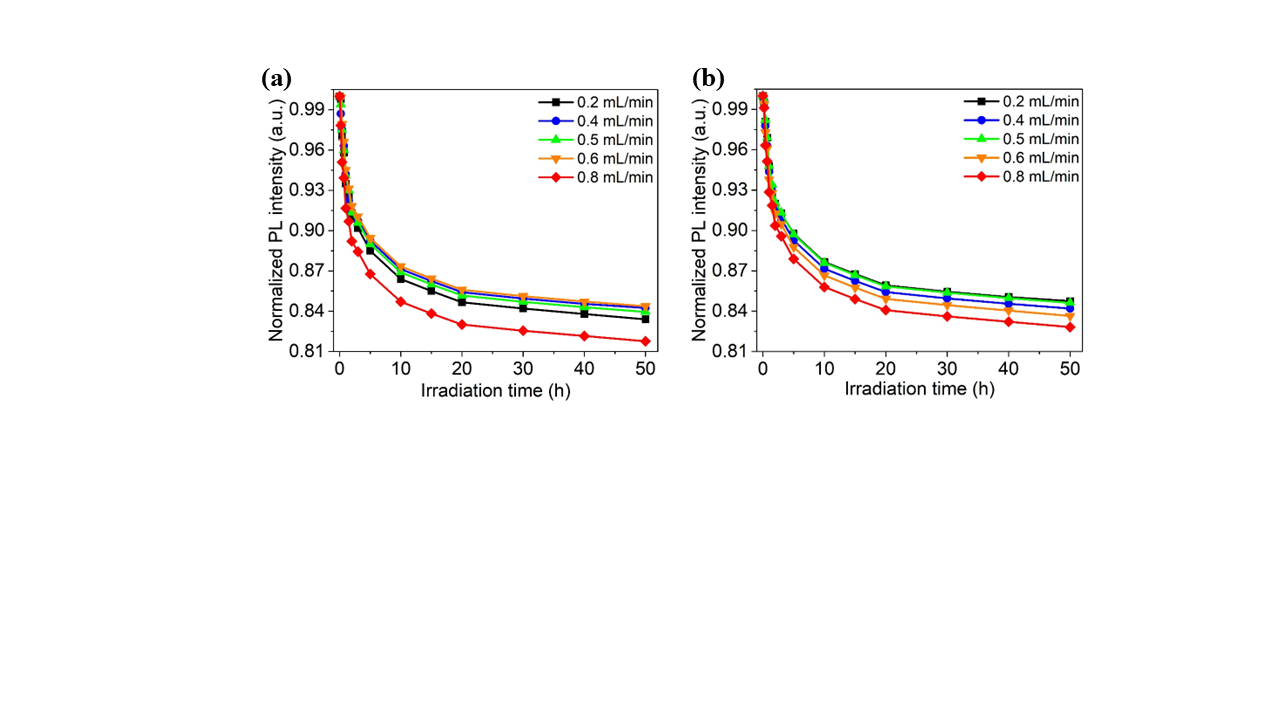


**Fig. S10**. Photostability of CDs synthesized at different flow velocities. (a) CDs obtained without ultrasonic treatment and (b) CDs obtained with ultrasonic treatment.

**Table S4.** The FWHM and peak position change of CDs excited with different wavelengths (360-480 nm) at various flow rates without ultrasonic treatment.

| **Flow velocity** | **FWHM/Peak redshift (nm)** | **360 nm EX** | **380 nm EX** | **400 nm EX** | **420 nm EX** | **440 nm EX** | **460 nm EX** | **480 nm EX** |
| --- | --- | --- | --- | --- | --- | --- | --- | --- |
| 0.2 mL/min | FWHM | 77.2  ± 1.5 | 80.4  ± 1.6 | 99.3  ± 2.5 | 126.2  ± 3.0 | 129.4  ± 3.0 | 105.1  ± 2.6 | 85.7  ± 3.0 |
|  | Redshift | 0 (Ref.) | 5 | 7 | 83 | 104 | 117 | 127 |
| 0.4 mL/min | FWHM | 80.3  ± 1.5 | 86.4  ± 1.6 | 102.9  ± 2.4 | 118.3  ± 2.5 | 112.1  ± 2.8 | 110.2  ± 2.5 | 93.4  ± 2.0 |
|  | Redshift | 0 (Ref.) | 6 | 27 | 79 | 99 | 114 | 128 |
| 0.5 mL/min | FWHM | 77.4  ± 1.4 | 81.1  ± 1.6 | 123.5  ± 2.5 | 112.5  ± 2.3 | 105.6  ± 2.0 | 112.5  ± 2.5 | 98.7  ± 2.0 |
|  | Redshift | 0 (Ref.) | 3 | 25 | 83 | 93 | 101 | 123 |
| 0.6 mL/min | FWHM | 75.4 ± 1.5 | 76.4  ± 1.6 | 89.4  ± 1.6 | 135.9  ± 3.5 | 127.5  ± 2.6 | 96.7  ± 2.0 | 96.9  ± 2.0 |
|  | Redshift | 0 (Ref.) | 3 | 6 | 75 | 92 | 104 | 123 |
| 0.8 mL/min | FWHM | 76.4  ± 1.5 | 77.3  ± 1.5 | 85.1  ± 1.6 | 126.4  ± 3.0 | 124.6  ± 3.0 | 118.3  ± 2.5 | 100.2  ± 2.0 |
|  | Redshift | 0 (Ref.) | 2 | 13 | 61 | 85 | 104 | 134 |


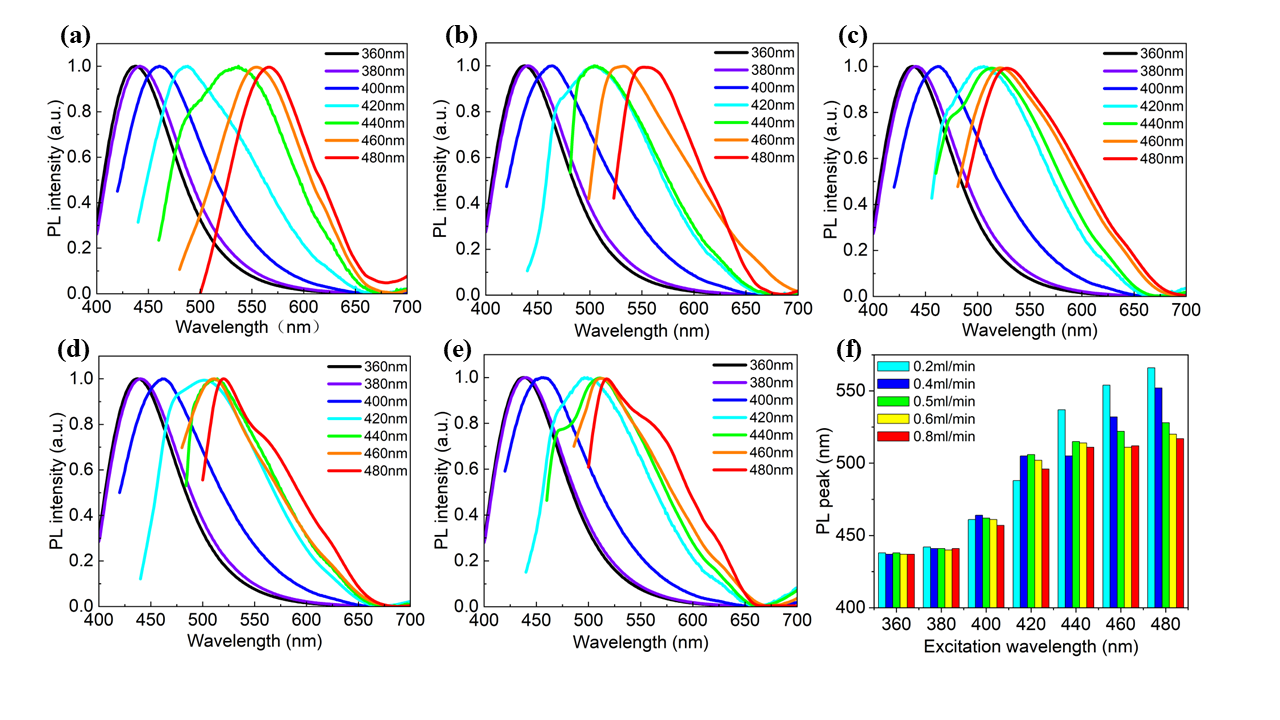


**Fig. S11.** (a) PL spectra and PL peak positions of CDs excited with different wavelengths (360-480 nm) at various flow rates with ultrasonic treatment: (a) 0.2 mL/min, (b) 0.4 mL/min, (c) 0.5 mL/min, (d) 0.6 mL/min, (e) 0.8 mL/min, (f) PL peak positions.

**Table S5.** The FWHM and peak position change of CDs excited with different wavelengths (360-480 nm) at various flow rates with ultrasonic treatment.

| **Flow velocity** | **FWHM/Peak redshift (nm)** | **360 nm EX** | **380 nm EX** | **400 nm EX** | **420 nm EX** | **440 nm EX** | **460 nm EX** | **480 nm EX** |
| --- | --- | --- | --- | --- | --- | --- | --- | --- |
| 0.2 mL/min | FWHM | 78.0  ± 1.5 | 82.3  ± 1.6 | 92.4  ± 2.0 | 113.1  ± 2.5 | 122.9  ± 3.0 | 96.8  ± 2.0 | 90.1  ± 2.0 |
|  | Redshift | 0 (Ref.) | 4 | 23 | 50 | 99 | 116 | 128 |
| 0.4 mL/min | FWHM | 77.9  ± 1.5 | 81.8  ± 1.6 | 101.9  ± 2.2 | 112.3  ± 2.5 | 94.6  ± 2.0 | 101.4  ± 2.3 | 87.1  ± 1.8 |
|  | Redshift | 0 (Ref.) | 4 | 27 | 68 | 69 | 95 | 115 |
| 0.5 mL/min | FWHM | 78.3  ± 1.5 | 82.1  ± 1.6 | 98.9  ± 2.0 | 114.0  ± 2.3 | 122.3  ± 3.0 | 116.9  ± 2.5 | 114.6  ± 2.5 |
|  | Redshift | 0 (Ref.) | 3 | 24 | 71 | 77 | 85 | 97 |
| 0.6 mL/min | FWHM | 77.9  ± 1.5 | 81.3  ± 1.6 | 99.1  ± 2.0 | 114.5  ± 2.5 | 91.7  ± 2.0 | 94.9  ± 2.0 | 92.9  ± 2.0 |
|  | Redshift | 0 (Ref.) | 3 | 24 | 65 | 77 | 78 | 83 |
| 0.8 mL/min | FWHM | 77.8  ± 1.5 | 79.9  ± 1.5 | 91.6  ± 2.0 | 107.2  ± 2.0 | 114.2  ± 2.5 | 94.3  ± 2.0 | 96.7  ± 2.0 |
|  | Redshift | 0 (Ref.) | 4 | 20 | 62 | 74 | 75 | 80 |

**Table S6.** The fit residuals, reduced χ² chi-square (χ²_red_), and Akaike Information Criteria (AIC) values for each fitting curve.

| **External excitation** | **Flow velocities (mL/min)** | **Status (Fit converged.)** | **AIC** | **BIC** | **Adj. R-Square** | **Reduced Chi-Sqr (**χ²_red_**)** |
| --- | --- | --- | --- | --- | --- | --- |
| Without ultrasonic treatment | 0.2 | 100 | -17327.6 | -17283.3 | 0.9935 | 1.06E-4 |
|  | 0.5 | 100 | -17818.0 | -17773.7 | 0.9921 | 8.69E-5 |
|  | 0.8 | 100 | -17637.7 | -17593.4 | 0.9947 | 9.45E-5 |
| With  ultrasonic treatment | 0.2 | 100 | -17485.0 | -17440.6 | 0.9903 | 1.07E-4 |
|  | 0.5 | 100 | -16910.8 | -16866.5 | 0.9918 | 1.36E-4 |
|  | 0.8 | 100 | -17373.9 | -17329.6 | 0.9925 | 1.13E-4 |


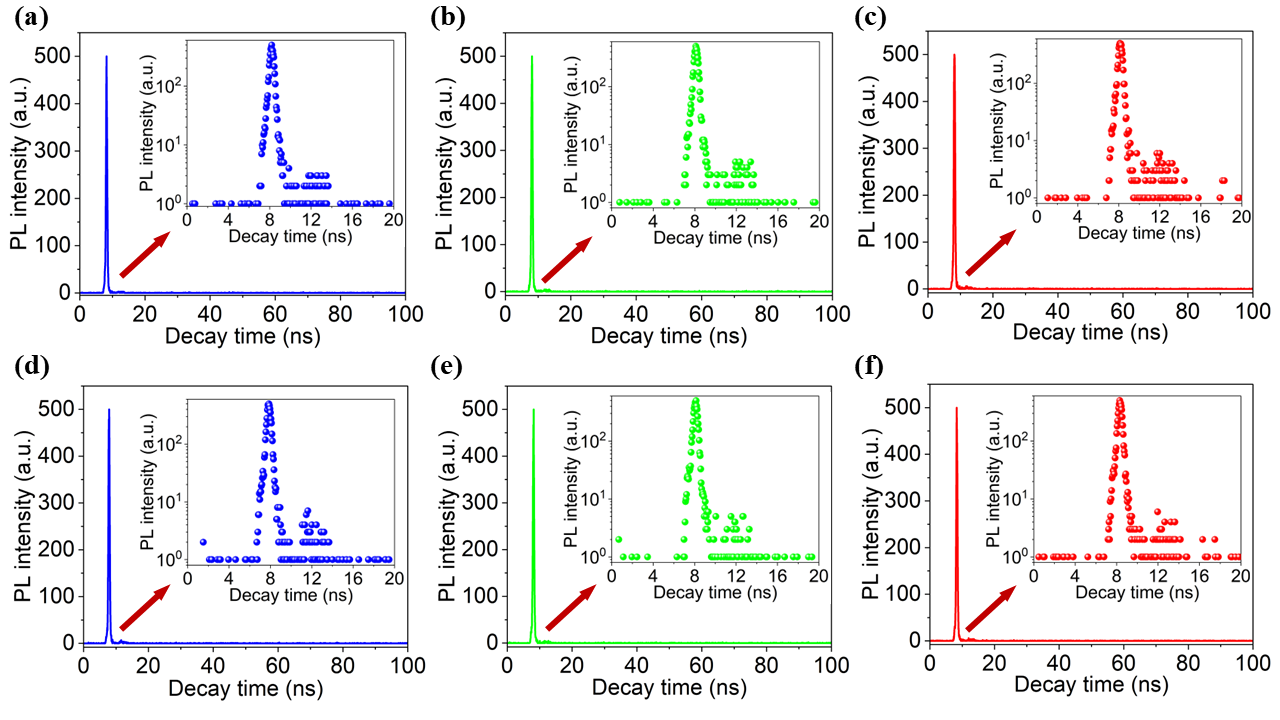


**Fig. S12**. CDs IRF curve at different flow velocities without ultrasonic treatment: (a) 0.2 mL/min, (b) 0.5 mL/min, (c) 0.8 mL/min. CDs IRF curve at different flow velocities with ultrasonic treatment: (d) 0.2 mL/min, (e) 0.5 mL/min, (f) 0.8 mL/min.


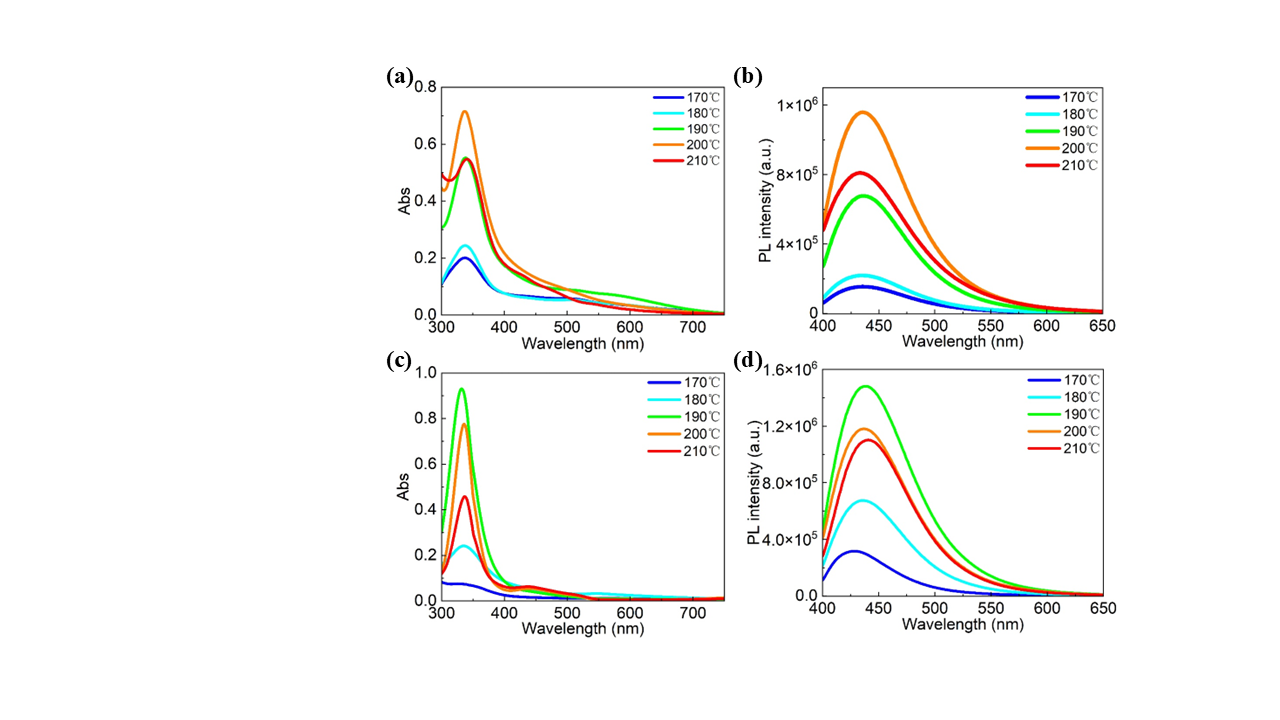


**Fig. S13.** (a) UV-vis absorption and (b) PL spectra of CDs synthesized at different reaction temperatures, with no ultrasonic treatment applied. (c) UV-vis absorption and (d) PL spectra of CDs synthesized at reaction temperatures after ultrasonic treatment.


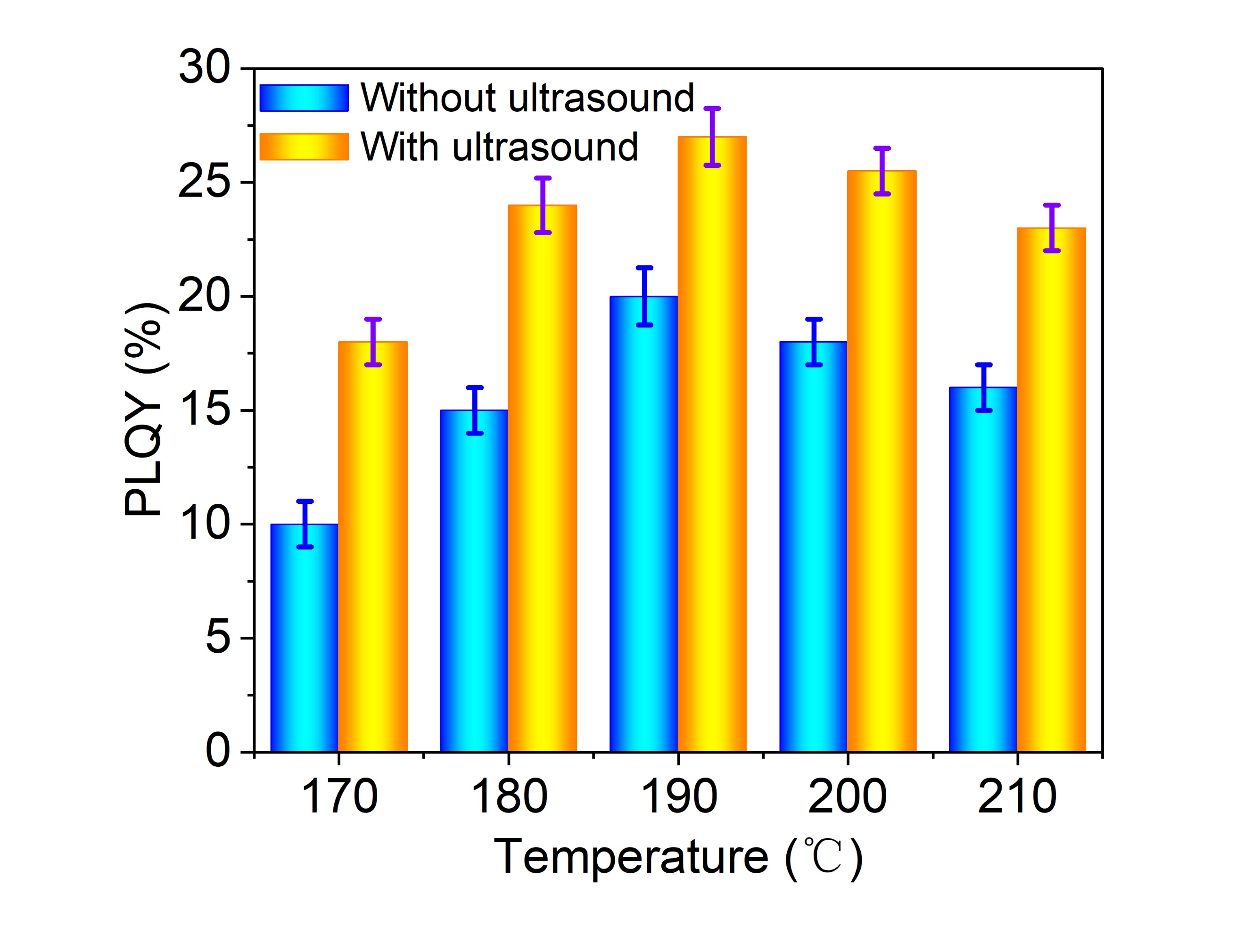


**Fig. S14.** Comparison of the PLQY for CDs synthesized under different conditions.

**Table S7.** The FWHM and peak position change of CDs synthesized under different conditions.

| **External excitation** | **Temperature (°C)** | **FWHM/**  **Peak redshift** | **360nm EX** | **380nm EX** | **400nm EX** | **420nm EX** | **440nm EX** | **460nm EX** | **480nm EX** |
| --- | --- | --- | --- | --- | --- | --- | --- | --- | --- |
| **Without ultrasonic treatment** | 170 | FWHM  (nm) | 79.9  ± 1.5 | 81.1  ± 2.2 | 82.3  ± 2.4 | 156.8 ± 3.5 | 101.9 ± 2.4 | 103.5 ± 2.6 | 133.8 ± 3.0 |
|  |  | Redshift  (nm) | 0 (Ref.) | 3 | 7 | 30 | 86 | 87 | 99 |
|  | 180 | FWHM  (nm) | 80.0  ± 1.5 | 82.2  ± 1.5 | 102.0 ± 2.8 | 132.9 ± 3.0 | 103.1 ± 2.5 | 101.7 ± 2.5 | 95.2  ± 2.0 |
|  |  | Redshift  (nm) | 0 (Ref.) | 3 | 25 | 80 | 92 | 97 | 126 |
|  | 190 | FWHM  (nm) | 80.2 ± 1.5 | 81.5  ± 1.6 | 90.5  ± 1.5 | 131.5 ± 3.0 | 115.6  ± 2.5 | 115.3  ± 2.5 | 95.3  ± 1.8 |
|  |  | Redshift  (nm) | 0 (Ref.) | 7 | 22 | 75 | 100 | 116 | 127 |
|  | 200 | FWHM  (nm) | 81.3  ± 1.5 | 82.4  ± 1.6 | 100.4  ± 2.0 | 142.4 ± 3.0 | 127.5  ± 2.6 | 124.9  ± 2.5 | 92.6  ± 1.5 |
|  |  | Redshift  (nm) | 0 (Ref.) | 9 | 21 | 70 | 105 | 118 | 125 |
|  | 210 | FWHM  (nm) | 81.6  ± 1.4 | 82.0  ± 1.5 | 102.6  ± 2.0 | 132.4 ± 3.0 | 130.4  ± 2.6 | 130.9  ± 2.5 | 93.9  ± 1.5 |
|  |  | Redshift  (nm) | 0 (Ref.) | 9 | 21 | 68 | 100 | 113 | 128 |
| **With**  **ultrasonic treatment** | 170 | FWHM  (nm) | 63.4  ± 1.0 | 63.1  ± 1.0 | 58.3  ± 1.0 | 105.4 ± 2.0 | 114.3  ± 2.4 | 72.8  ± 1.6 | 80.8  ± 1.8 |
|  |  | Redshift  (nm) | 0 (Ref.) | 2 | 8 | 58 | 83 | 109 | 134 |
|  | 180 | FWHM  (nm) | 75.3  ± 1.5 | 74.9  ± 1.5 | 66.8  ± 1.0 | 98.9  ± 2.0 | 105.1 ± 2.5 | 162.5 ± 3.0 | 160.2  ± 3.0 |
|  |  | Redshift  (nm) | 0 (Ref.) | 3 | 6 | 21 | 62 | 83 | 129 |
|  | 190 | FWHM  (nm) | 79.7  ± 1.5 | 80.7  ± 1.6 | 86.0  ± 1.5 | 98.4 ± 2.0 | 117.5  ± 2.5 | 115.4  ± 2.5 | 106.1  ± 2.2 |
|  |  | Redshift  (nm) | 0 (Ref.) | 1 | 25 | 49 | 83 | 87 | 121 |
|  | 200 | FWHM  (nm) | 77.6  ± 1.5 | 80.9  ± 1.6 | 91.9  ± 2.0 | 124.8 ± 2.0 | 125.3  ± 2.5 | 97.5  ± 2.0 | 89.5  ± 1.5 |
|  |  | Redshift  (nm) | 0 (Ref.) | 7 | 22 | 57 | 109 | 122 | 131 |
|  | 210 | FWHM  (nm) | 76.6  ± 1.5 | 76.0  ± 1.5 | 79.8  ± 1.5 | 133.9 ± 3.0 | 122.9  ± 2.6 | 94.3  ± 2.0 | 90.5  ± 1.5 |
|  |  | Redshift  (nm) | 0 (Ref.) | 5 | 12 | 45 | 111 | 119 | 126 |
